# An integrated anti-aging framework targeting NAD^+^ homeostasis, mitochondrial quality control, and redox stability: Roles of NMN/NR, PQQ, and EGT

**DOI:** 10.1016/j.redox.2026.104191

**Published:** 2026-04-24

**Authors:** Yidan Sun, June-Chiew Han, Kenneth Tran, Toan Pham, Qiang Zhou, Jun Lu

**Affiliations:** a Auckland Bioengineering Institute, University of Auckland, Auckland, 1142, New Zealand; b Department of Endocrinology, Affiliated Hospital of Jiaxing University (The First Hospital of Jiaxing), Jiaxing, 314001, China; c School of Chemical Sciences, University of Auckland, Auckland, 1142, New Zealand; d Future Food and Agriculture Department, Yangtze Delta Region Institute of Tsinghua University, Jiaxing, Zhejiang, 314006, China

**Keywords:** NMN, NR, PQQ, Ergothioneine, Anti-aging, Mitochondria

## Abstract

As the global population ages rapidly, delaying and preventing age-related diseases have become urgent priorities in public health and biomedical research. During aging, mitochondrial dysfunction is a core molecular hallmark and a common pathogenic mechanism underlying multiple age-related disorders. Age-related mitochondrial dysfunction typically manifests as diminished metabolic capacity, impaired organelle renewal, and disrupted redox homeostasis. These factors interact to form a feedback loop constraining mitochondrial adaptability. Specifically, the interdependent decline in NAD^+^ availability, impaired mitochondrial biogenesis, and excessive oxidative stress render single-pathway interventions ineffective in mitigating systemic functional impairments triggered by aging.

To address this complex mechanism, this review presents a novel tri-axis anti-aging model encompassing three key compounds: nicotinamide mononucleotide/nicotinamide riboside (NMN/NR), pyrroloquinoline quinone (PQQ), and l-ergothioneine (EGT). Within this framework, NMN/NR serves as a broad NAD^+^-dependent regulator of mitochondrial homeostasis, with its most immediate effects on metabolic activation, while PQQ and EGT may further strengthen mitochondrial remodeling and redox resilience, respectively. While each compound has distinct functional emphases, they are highly mechanistically coupled, collectively forming a closed-loop network regulating mitochondrial number, function, and homeostasis. This review synthesizes preclinical and emerging clinical evidence supporting the standalone or combined use of NMN/NR, PQQ, and EGT across various diseases.

Collectively, by conceptualizing mitochondrial aging as a systemic imbalance rather than isolated molecular defects, this paper highlights a three-axis model of NMN/NR, PQQ, and EGT. This framework offers a theoretical foundation for mitochondrial-targeted anti-aging interventions while laying the groundwork for future clinical research, nutritional interventions, and the development of multi-target combination strategies.

## Highlights

• Presented a comprehensive review of NMN/NR, PQQ, and EGT in aging.
• Suggested a tri-axis mitochondrial model for anti-aging regulation.
• Discussed the possible combination of NMN/NR, PQQ, and EGT in anti-aging applications.

## Graphical abstract

**Figure:**
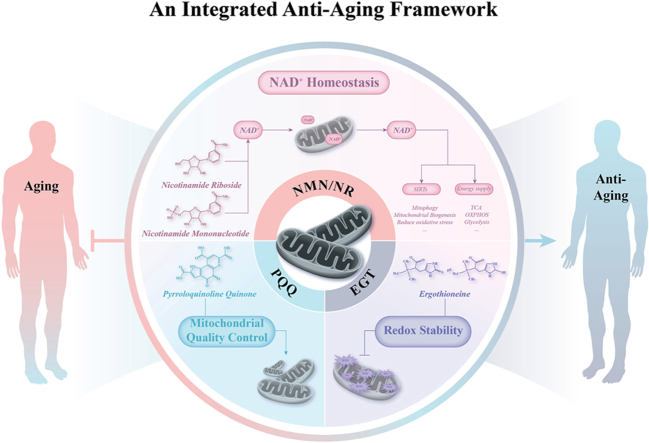

## 1 Introduction

**Fig. 1:**
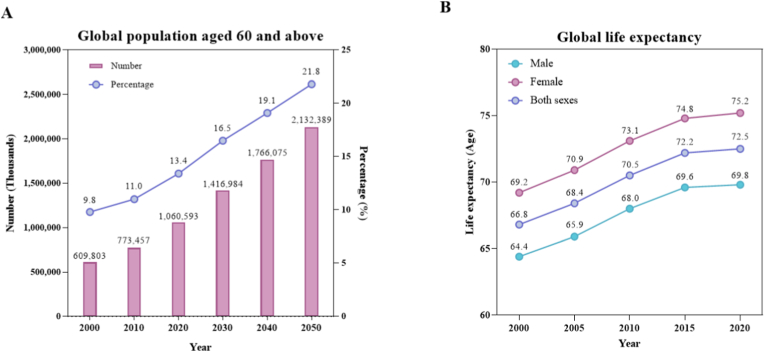
**Global aging population in recent years.** A: Global Population Aged 60 and Over and Related Percentage; B: Global Life Expectancy. The graph was constructed based on data from the Data portal of WHO (Number of persons aged over 60 years or over [1]; Life expectancy at birth [2]).

Global population aging has intensified interest in understanding the aging process and in developing strategies and interventions to extend health span. The accelerating pace of global population aging, as reported by the World Health Organization (WHO), is shown in Fig. 1. Accordingly (Fig. 1A), by 2030, one in six people worldwide will be 60 years or older, and by 2050, it is expected to reach approximately 2.1 billion, i.e., one in five people [3]. In addition, global life expectancy has increased from 60 to 70 years for both males and females over the last 20 years (Fig. 1B). In addition to the demographic transition, population aging imposes substantial socioeconomic challenges on healthcare systems and societal structures worldwide. Aging is closely associated with a high incidence of multiple chronic diseases, including congestive heart failure, myocardial infarction, dementia, stroke, most cancers, diabetes and metabolic disorders, renal dysfunction, chronic lung disease, osteoporosis, arthritis, and vision loss [4]. The greatest clinical challenge in treating the growing number of elderly patients is multimorbidity; at least half of adults aged 70 and older have multimorbidity, along with the use of five or more medications (polypharmacy). This situation occurs in more than 10% of the general population, affecting more than 10% of adults and 30% of older adults [5].

In the World Report on Ageing and Health, the WHO proposed a public health framework in 2015 that includes strategies for health services, long-term care, and age-friendly environments [6]. Different countries are adopting diverse strategies to address these challenges. Some countries have increasingly emphasized healthy aging and preventive healthcare, aiming to extend health span rather than lifespan alone. Policy frameworks such as integrated care systems, community-based long-term care, and age-friendly environments have been implemented to reduce healthcare burden and improve quality of life. For example, over the past 20 years, Chinese aging policies have been characterized by a focus on centralized, comprehensive governance and top-level design, while also encouraging local innovation in areas such as integrated healthcare, long-term care, and smart aging [7]. Countries with limited healthcare infrastructure face additional challenges in balancing aging-related care demands with economic development. Research suggests that the country's healthcare system must be reformed to focus on expanding access to critical care, particularly for the elderly and palliative care, in order to meet the healthcare needs arising from these demographic changes [8].

These global trends highlight that aging is not only a biological process but also a complex societal issue, reinforcing the urgent need for effective intervention strategies targeting fundamental mechanisms of aging. In addition to dietary interventions and physical exercise, pharmacological approaches represent one of the most promising strategies for targeting aging and age-related diseases and have been incorporated into research programs by the National Institute on Aging [9]. Currently, many small molecules have emerged as candidates for delaying human aging, preventing the onset and/or progression of diseases, and maintaining human functional capacity in later life [10].

The development of aging phenotypes and the increase in biological age are driven by complex and multifactorial mechanisms that are highly interconnected at the molecular level [11]. In 2023, López-Otín et al. expanded the hallmarks of aging from the original nine proposed in 2013 [12] to twelve criteria, including: genomic instability, telomere attrition, epigenetic alterations, loss of proteostasis, disabled macroautophagy, deregulated nutrient sensing, mitochondrial dysfunction, cellular senescence, stem cell exhaustion, altered intercellular communication, chronic inflammation, and dysbiosis [13]. Notably, both the original and the revised frameworks identify mitochondrial dysfunction as a core molecular feature of physiological aging, and mitochondrial impairment can actively drive the aging process by influencing other hallmarks [14]. For this reason, mitochondrial dysfunction has emerged as a critical therapeutic target in the development of strategies to mitigate aging phenotypes and age-related diseases [15].

Mitochondria are multifunctional organelles that play a crucial role in cellular energy metabolism, generating most cellular ATP through oxidative phosphorylation (OXPHOS) [16]. In aging, mitochondrial function declines due to the accumulation of mitochondrial DNA (mtDNA) mutations, instability of respiratory chain complexes, and altered mitochondrial dynamics [17]. The resulting massive production of reactive oxygen species (ROS) induces damage that further amplifies mitochondrial dysfunction, creating a self-reinforcing cycle[18]. Research on mitophagy-related pathways such as Pink1 [19] and PGC-1α [20] suggests that aging impairs mitochondrial clearance and recycling, allowing defective mitochondria to persist and thereby exacerbating neurodegeneration and inflammation. A variety of compounds, including mitohormetins and inhibitors of the mitochondrial electron transport chain, mitophagy inducers, PPAR and PGC-1α activators, mitoproliferation activators, and NAD^+^ precursors have been investigated to modulate aging by targeting mitochondrial function [21]. However, most studies focus on isolated mechanisms and lack an integrated framework. This gap limits our understanding of the overall efficacy of mitochondria-targeted anti-aging interventions.

A structured literature search was conducted using PubMed, Web of Science, and Scopus to identify studies related to mitochondrial dysfunction, aging mechanisms, and mitochondria-targeted interventions. Search terms included combinations of “mitochondrial dysfunction”, “aging”, “mitochondrial biogenesis”, and “NAD^+^ metabolism”. Studies were screened for relevance to mitochondrial regulatory pathways implicated in aging, including mitochondrial energy metabolism, mitochondrial quality control, and redox homeostasis. To construct an integrated conceptual framework, the selected literature was compared to identify key molecular regulators that target distinct yet complementary aspects of mitochondrial aging. Particular attention was given to compounds with substantial experimental evidence demonstrating their ability to modulate mitochondrial metabolism, mitochondrial biogenesis and quality control, or mitochondrial redox stability.

Based on an integrated analysis of the literature, this review suggests a mitochondria-targeted anti-aging strategy organized into three regulatory axes, nicotinamide mononucleotide/nicotinamide riboside (NMN/NR), pyrroloquinoline quinone (PQQ), and ergothioneine (EGT). NMN is a bioactive nucleotide composed of nicotinamide, ribose, and a phosphate group [22]. They serve as key precursors and intermediate in nicotinamide adenine dinucleotide (NAD^+^) biosynthesis, and dietary NMN/NR supplementation has been shown to increase intracellular NAD^+^ levels, which may help reverse age-related mitochondrial decline [23]. PQQ regulates mitochondrial physiology and biogenesis and acts as an essential cofactor in many biological processes [24]. EGT, a sulfur-containing amino acid derived from histidine, is capable of scavenging harmful free radicals, including reactive oxygen species and reactive nitrogen species [25]. A specific transporter for EGT is highly enriched in mitochondria and has been shown to play a unique role in protecting mitochondrial components from oxidative damage associated with mitochondrial superoxide production [26].

**Fig. 2:**
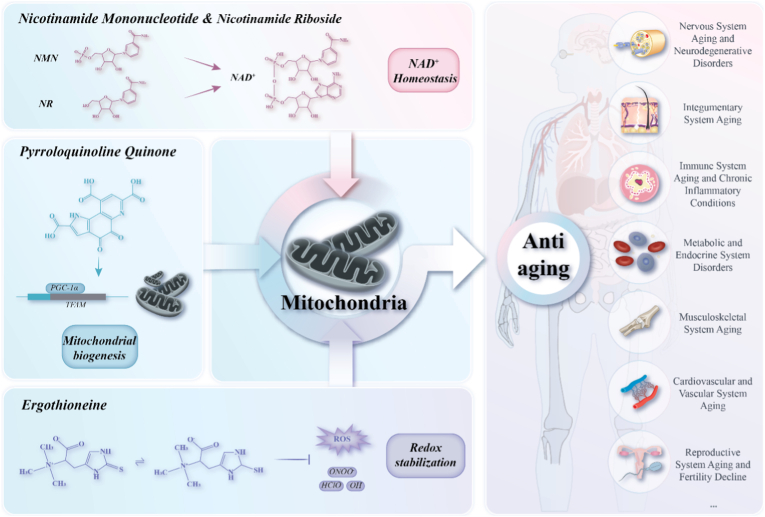
**The presented anti-aging framework: Roles of NMN/NR, PQQ, and EGT.** This figure illustrates the proposed tri-axis framework as a model of functional emphasis. NMN/NR, PQQ, and EGT are presented as representative modulators with predominant effects on metabolic activation, mitochondrial remodeling, and redox buffering, respectively. NAD^+^: Nicotinamide adenine dinucleotide (oxidized); ROS: reactive oxygen species; ONOO^−^: Peroxynitrite; HOCl: Hypochlorous acid; ·OH: Hydroxyl radical.

This review suggests a coordinated anti-aging framework which is shown in Fig. 2 consisting of three regulatory dimensions: metabolic activation, mitochondrial remodeling, and redox buffering. Within this framework, NMN/NR, PQQ, and EGT are discussed as representative compounds with different functional emphases rather than mutually exclusive roles. NMN/NR exerts broad effects across mitochondrial metabolism, quality control, and stress adaptation, whereas PQQ and EGT may further strengthen these processes by enhancing mitochondrial remodeling capacity and redox resilience, respectively. This anti-aging model is expected to be applied in combination with treatments for various age-related diseases.

## 2 NAD ^+^ homeostasis in aging and NMN/NR

### 2.1 NAD ^+^ depletion during aging

NAD^+^ is an essential coenzyme in cellular redox reactions and a central regulator of energy metabolism [27]. With age, intracellular NAD^+^ levels decline progressively, a phenomenon that is now recognized as one of the key molecular features of aging. This decline arises from two major mechanisms. First, NAD^+^ biosynthetic capacity decreases, particularly due to reduced expression or activity of the rate-limiting enzyme nicotinamide phosphoribosyltransferase (NAMPT). Second, the accumulation of DNA damage and chronic inflammation leads to increased activity of NAD^+^-consuming enzymes, including poly (ADP-ribose) polymerases (PARPs) and CD38, thereby accelerating NAD^+^ depletion [28]. NAD^+^ levels not only directly regulate cellular energy metabolism but also indirectly influence mitochondrial function. Studies have shown that enhancing NAD^+^ availability improves mitochondrial performance under stress conditions [29]. NAD^+^ links tricarboxylic acid (TCA) cycle intermediates to the electron transport chain (ETC) and oxidative phosphorylation-driven adenosine triphosphate (ATP) production, while activating sirtuin proteins (such as SIRT3 and SIRT5) to optimize mitochondrial metabolism.

In addition to its role in metabolic regulation, emerging evidence suggests that NAD^+^ availability is closely linked to mitochondrial quality control through the regulation of mitophagy. Aging-associated NAD^+^ decline has been proposed to impair the NAD^+^ mitophagy axis, thereby contributing to the accumulation of dysfunctional mitochondria and increasing susceptibility to neurodegenerative processes [30]. This concept has also been supported by multiple preclinical studies [[31], [32], [33]]. Premature aging disorders further support the role of impaired NAD^+^-dependent mitochondrial quality control. Diseases such as Werner syndrome [31,32], ataxia-telangiectasia[34], and xeroderma pigmentosum [35] exhibit features of accelerated aging and are consistently associated with mitochondrial dysfunction and defective mitophagy. Importantly, emerging evidence indicates that restoration of mitophagy can alleviate disease phenotypes in these conditions, as demonstrated in both preclinical models and early clinical observations [36]. These observations further indicate that age-associated NAD^+^ decline may impair mitochondrial quality control not only by limiting metabolic regulation but also by compromising the selective clearance of damaged mitochondria, thereby contributing to the progressive accumulation of mitochondrial dysfunction. Accordingly, restoration of NAD^+^ homeostasis may influence not only bioenergetic capacity but also mitochondrial quality control and stress adaptation during aging. Therefore, restoring age-associated NAD^+^ deficiency is considered highly important for delaying, or even reversing, the progression of aging-related diseases.

NAD^+^ homeostasis is maintained through three distinct biosynthetic pathways: the de novo pathway, the Preiss-Handler pathway, and the salvage pathway [27]. The major NAD^+^ precursors in different biosynthetic pathways include tryptophan (Trp), nicotinic acid (NA), nicotinamide (NAM), and nicotinamide riboside (NR) [37]. Among these, Trp is primarily utilized for protein synthesis and other biosynthetic processes, and its efficiency for NAD^+^ biosynthesis is approximately 60-fold lower than that of NA [38]. In addition, mammalian tissues have limited exposure to NA, which typically circulates at low plasma concentrations [39], and NA can be rapidly converted to NAM in the intestine and liver [40]. These factors suggest that the plasma levels of most NAD^+^ precursors may be insufficient to sustain high systemic rates of NAD^+^ production. Interestingly, intermediate compounds in the NAD^+^ biosynthetic pathways, such as NMN/NR, can directly stimulate NAD^+^ synthesis via both the salvage and Preiss-Handler pathways.

### 2.2 NMN/NR and its anti-aging effects

**Fig. 3:**
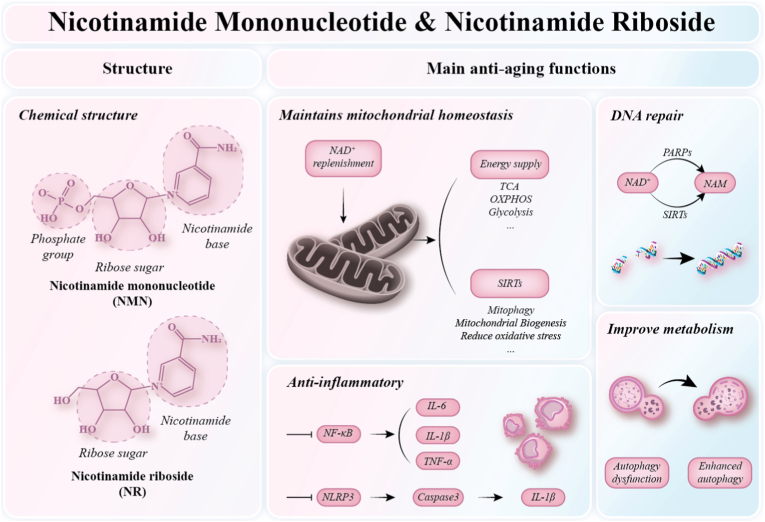
**The Chemical structure and main anti-aging functions of NMN/NR** NAD^+^: Nicotinamide adenine dinucleotide (oxidized); TCA: Tricarboxylic acid cycle; OXPHOS: Oxidative phosphorylation; NAM: Nicotinamide; PARP1: Poly(ADP-ribose) polymerase 1; SIRT: Sirtuin; NF-κB: Nuclear factor κB; IL-6: Interleukin-6; IL-1β: Interleukin-1β; TNF-α: Tumor necrosis factor-α; NLRP3: NOD-like receptor family pyrin domain-containing 3.

NMN is synthesized by nicotinamide NAMPT from NAM, a water-soluble form of vitamin B_3_, and 5′-phosphoribosyl-1-pyrophosphate (PRPP) [41]. It is a bioactive nucleotide containing a pyridine base. NR is an individual chemical moiety consisting of nicotinamide and ribose. NMN and NR are both key intermediates in the synthesis of NAD^+^, and they have molecular weights of 334.22 g/mol [42] and 255.25 g/mol [43], respectively. Structural formulas of NMN and NR are shown in Fig. 3. NMN is naturally present in a variety of plant- and animal-derived foods [44], including edamame, broccoli, cherry blossoms, avocado, mushrooms, beef, and shrimp [45]. The earliest reports on NMN date back to 1963 [46], and since then, interest in NMN/NR as an anti-aging agent has gradually emerged. With aging, circulating levels of extracellular nicotinamide phosphoribosyltransferase (eNAMPT) decline, leading to a systemic reduction in NAD^+^ levels [47]. Multiple studies have demonstrated that NMN/NR supplementation effectively enhances NAD^+^ biosynthesis in various peripheral tissues, including adipose tissue [48], brain [49], skeletal muscle [50], eye [51], and vasculature [52] and alleviates age-associated physiological decline in rodent models, thereby exerting therapeutic effects on aging-related disorders [31,32,45,53,54]. Collectively, NMN/NR acts as a broad regulator of mitochondrial homeostasis by restoring NAD^+^ availability. Its most immediate effect is metabolic, as NAD^+^ repletion supports glycolysis, fatty acid oxidation, the TCA cycle, and oxidative phosphorylation. However, the effects of NMN/NR extend beyond energy supply alone. Through sirtuin-dependent pathways, NMN/NR may also influence mitochondrial quality control, biogenesis-related signaling, antioxidant defense, and mitophagy-associated processes.

NMN/NR efficiently enters cells and distributes throughout tissues, demonstrating proven anti-aging effects. Its anti-aging effects are centered on NAD^+^ restoration, but the downstream consequences include broader regulation of mitochondrial metabolism, stress resistance, and quality control. In addition, it also exhibits DNA repair, enhanced metabolism, and anti-inflammatory effects (Fig. 3).

Previous studies have shown that NMN/NR supplementation leads to a significant restoration of intracellular NAD^+^ levels, particularly within mitochondrial-enriched fractions, a process that depends on mitochondrial-associated nicotinamide mononucleotide adenylyltransferases (NMNATs) to catalyze NAD^+^ resynthesis [55]. Unlike NAD^+^ itself, NMN can bypass the limitations of NAD^+^ transmembrane transport by locally replenishing mitochondrial NAD^+^ pools, functionally compensating for the inability of NAD^+^ to be directly transported into mitochondria [56]. The effects of NMN/NR extend beyond its role as a simple NAD^+^ “precursor.” Restoration of NAD^+^ availability reactivates a series of NAD^+^-dependent deacetylases, particularly sirtuins such as SIRT1, SIRT3, and SIRT5. These enzymes finely regulate oxidative phosphorylation, fatty acid oxidation, and antioxidant defense systems by driving the deacetylation of mitochondrial proteins [27]. Accumulating evidence indicates that NMN-mediated sirtuin activation increases mitochondrial resilience under metabolic and oxidative stress conditions [57,58]. NMN rescues TDP-43 deficiency-induced cell death by inhibiting the mitochondrial α-ketoglutarate-dependent dioxygenase ALKBH7 [59]. Research indicates that when NAD^+^ levels are insufficient, SIRT1 fails to inhibit hypoxia-inducible factor 1α (HIF-1). Elevated HIF-1 levels disrupt communication between mitochondria and the nucleus at the cellular level, and between adipose tissue and the hypothalamus at the systemic level. This disruption in mitochondrial-nuclear communication accelerates mitochondrial dysfunction, subsequently triggering age-related complications and disease progression. However, NMN/NR, acting as an NAD^+^ precursor [50], restores specific communication functions and mitochondrial health. In mouse models of Alzheimer's disease, NMN/NR supplementation alleviates mitochondrial proteotoxicity, reduces synaptic damage and hippocampal neuronal loss, suppresses brain atrophy, and delays disease progression [60].

Furthermore, aging induces other biological changes that can also be prevented by increasing the body's NAD^+^ levels through NMN/NR supplementation. Members of the PARP family are crucial in NAD^+^-mediated DNA damage repair. PARP-3 primarily participates in repairing double-strand DNA breaks, while PARP-1 and PARP-2 are activated in base excision repair triggered by single-strand DNA breaks [61]. PARP and sirtuin families exhibit mutual dependence and regulation during DNA repair, working synergistically. When NAD^+^ is depleted, SIRT1 is activated, directly mediating apoptosis [35].

NMN/NR activates autophagy-related signaling pathways (such as SIRT1). SIRT1 activation stimulates autophagy-related gene expression and enhances autophagy activity. NMN/NR also contributes to the regulation of mitophagy, thereby supporting mitochondrial quality control and turnover. This enables cells to more efficiently clear waste and damaged components, thereby maintaining cellular health [62]. With aging, cellular autophagy gradually declines, leading to the accumulation of intracellular waste and functional deterioration. NMN/NR delays cellular aging by enhancing autophagy. Intraperitoneal injection or oral administration of NMN increases the release of anti-inflammatory cytokines while reducing pro-inflammatory cytokines (H. [[63], [64], [65], [66]]). Research indicates that NMN/NR promotes AMP-activated protein kinase (AMPK) activation, thereby inhibiting nuclear factor kappa-B (NF-κB) signaling. This downregulates inflammatory factors, including IL-10, IL-6, and TNF-α [67].

### 2.3 Preclinical and emerging clinical evidence

NMN/NR has been investigated across multiple experimental systems, ranging from cellular models to animal studies and human clinical trials. These studies consistently demonstrate that NMN/NR supplementation elevates NAD^+^ levels and influences metabolic and mitochondrial regulatory pathways. The representative models used in NMN/NR research are summarized in Table 1.

**Table 1:** **Experimental model evidence for NMN/NR** IP: Intraperitoneal injection; PO: Per os (oral administration).

Study model	Gender	Age/Weight	Route	Dose	Major findings	Reference
HS68			Add to the culture medium	1ppm-100 ppm	NAD^+^↑; SIRT↑	[68]
Primary CMVECs			Add to the culture medium	5 × 10^-4 mol/L; for 1 to 5 days	NAD^+^↑; Angiogenic capacity↑	[69]
A549; SPCA1			Add to the culture medium	100 mM	SIRT1-AMPK-ACC ↑; Ferroptosis↑	[70]
MSCs			Add to the culture medium	100 μm for 48 h	NAD^+^↑; SIRT3↑	[65,66]
Wistar (albino)	Female	12-month-old (300–350 g); 5-month-old (200–220 g)	IP	500 mg/kg per day	SIRT1↑	[71]
C57BL/6J	Male	16-month-old	PO-water	500 mg/L (w/v) for 4 months	NAD^+^↑; SIRT3/6↑; TNF-α↓	[72]
C57BL/6J; 129Sv	Male	6-week-old to 10-11-week-old	IP	500 mg/kg, twice a week	SIRT3↑	[73]
Healthy adult human beings	Man and Woman	40-year-old to 65-year-old	PO	300 mg, 600 mg, and 900 mg per day for 60 days	NAD^+^↑;	[74]
Healthy adult human beings	Man and Woman	20-year-old to 65-year-old	PO	250 mg per day for 12 weeks	NAD^+^↑;	[75]
Healthy adult human beings	Man	Older than 60-year-old	PO	250 mg per day for 12 weeks	NAD^+^↑;	[76]

At the cellular level, NMN/NR is primarily studied for its effects on NAD^+^ levels, mitochondrial function, energy metabolism, and cellular stress states. Multiple studies indicate that NMN/NR effectively elevates intracellular NAD^+^ content across diverse cell types, thereby improving energy metabolism and enhancing cellular functional stability. For instance, in vascular endothelial cell models, NMN/NR treatment significantly ameliorates dysfunction induced by aging or stress, with its effects closely linked to restoration of NAD^+^ levels and activation of related metabolic pathways [69]. In human lung cancer cells A549, high doses of NMN/NR promote ferroptosis through the NAM-mediated SIRT1-AMPK-ACC signaling pathway [70]. In fibroblasts, NMN/NR elevates cellular NAD^+^ levels, activating sirtuin and autophagy pathways [68]. NMN/NR supplementation also influences mesenchymal stem cells (MSCs) by alleviating mitochondrial dysfunction through the NAD+/Sirt3 pathway, thereby rescuing cellular senescence [68]. NMN/NR supplementation also influences mesenchymal stem cells by alleviating mitochondrial dysfunction through the NAD^+^/Sirt3 pathway, thereby rescuing cellular senescence [65,66]. The study of NMN/NR in vivo models has primarily focused on age-related functional decline, metabolic disorders, and organ dysfunction. Multiple studies in rodents demonstrate that long-term or intermittent NMN supplementation significantly elevates tissue or systemic NAD^+^ levels. This finding aligns closely with cellular-level research outcomes and further substantiates improvements in systemic function. For instance, in natural aging mouse models, NMN/NR alleviates follicular development disorders by reducing ovarian hyperfollicularization in middle-aged rats and increasing SIRT1 activity, thereby enhancing reproductive-related functions [71]. In exogenously induced mouse models of aging, the sirtuin pathway can also be effectively activated by NMN/NR. Previous studies have demonstrated that NMN/NR reverses d-galactose-induced neurodegeneration by activating the SIRT3 pathway, thereby enhancing intestinal barrier function and slowing the aging process in mice [72]. In specific metabolic disorder models, NMN/NR 's effects also depend on the sirtuin signaling pathway. For example, in a Friedreich's ataxia cardiomyopathy model, NMN/NR requires SIRT3-mediated mechanisms to improve cardiac function and myocardial bioenergetic status [73]. Overall, animal studies consistently support NMN/NR as a NAD^+^-restoring intervention with broad effects on mitochondrial metabolism, quality control, and stress adaptation, although its most direct and best-established action remains metabolic.

In recent years, clinical research on NMN/NR has gradually accumulated preliminary evidence, primarily focusing on its safety, tolerability, and effects on metabolic and function-related indicators. Multiple studies involving healthy volunteers or specific populations indicate that oral NMN/NR is well-tolerated and safe within a specific dosage range. For example, research shows that daily oral administration of NMN/NR up to 900 mg remains safe and well-tolerated, while clinical benefits measured by blood NAD^+^ concentration and physical performance reach a relative peak at a daily dose of 600 mg [74]. While effectively increasing NAD^+^-related indicators in peripheral tissues or the circulatory system, no significant adverse reactions were observed in the studies [75]. Additionally, NMN/NR supplementation has been reported to effectively elevate NAD^+^ levels in middle-aged and elderly individuals or those with impaired metabolic function, thereby helping to prevent or alleviate age-related muscle dysfunction to a certain extent[76]. Overall, existing clinical evidence supports NMN/NR as an intervention molecule centered on metabolic support.

### 2.4 Limitations and cross-axis interaction

Although NMN/NR exerts broad effects on mitochondrial metabolism, redox-related signaling, and quality control pathways, these effects may not always be sufficient to fully restore mitochondrial homeostasis in the aging context. In particular, age-associated defects in mitochondrial remodeling capacity and impaired redox resilience may limit the durability of NAD^+^-driven metabolic improvement. Although a large body of clinical and animal studies generally indicates that NMN/NR is well tolerated in healthy adults and rodent models, with no obvious toxicity, this favorable safety profile does not imply that NMN/NR alone is sufficient as an anti-aging intervention across all aging contexts. NAD^+^ repletion enhances mitochondrial metabolic activity and energy production; however, mitochondrial respiration is also a major source of ROS [77]. Cellular ROS levels are determined by the balance between ROS generation and antioxidant defense systems. Therefore, increased metabolic flux places greater demand on redox regulation, and insufficient antioxidant buffering capacity may lead to oxidative imbalance. This highlights a conditional balance between enhanced energy metabolism and oxidative stress. For example, although oral NMN/NR administration does not show clear adverse effects, intraperitoneal NMN/NR injection under specific experimental conditions has been reported to increase oxidative stress levels in sperm tissue [78]. In addition, in specific cellular models, significant improvements in mitochondrial quality control and cell survival are observed only when NMN/NR supplementation is combined with inhibition of excessive NAD^+^ consumption pathways, such as with the PARP1 inhibitor PJ-34 [58,[79], [80], [81]].

Therefore, within the present framework, PQQ and EGT are not introduced because NMN/NR lacks relevance to mitochondrial remodeling or redox regulation, but because they may further reinforce these dimensions under conditions where NAD^+^ restoration alone is insufficient to sustain long-term mitochondrial adaptation.

## 3 Mitochondrial quality control in aging and PQQ

### 3.1 Mitochondrial quality control in aging

During aging, mitochondrial function declines systemically, a phenomenon highly consistent across multiple tissues and species. Mitochondrial morphology undergoes marked alterations, including abnormal mitochondrial rounding, a reduction in mitochondrial DNA content accompanied by an increased mutation rate, decreased respiratory chain activity, and impaired mitochondrial biogenesis, which refers to the process by which new mitochondria are generated from pre-existing mitochondria [82]. Impairment of mitochondrial biogenesis may slow the turnover of mitochondrial components, leading to the accumulation of oxidative damage to lipids, proteins, and DNA [83]. Although mitochondrial homeostasis is regulated through complex interactions among mitochondrial dynamics, mitochondrial biogenesis, and mitophagy [84], mitochondrial biogenesis functions as an upstream process that maintains mitochondrial renewal and functional supply. Its impairment is commonly associated with reduced adenosine triphosphate synthesis capacity and accelerated mitophagy [85]. Therefore, in aging cells, mitochondrial biogenesis dysfunction may represent a central event driving mitochondrial dysfunction. Delaying or reversing age-related declines in mitochondrial biogenesis is of critical importance for promoting healthy aging, preventing age-associated diseases, and improving quality of life in older individuals.

Despite the complexity of signaling pathways regulating mitochondrial biogenesis, they generally converge on the peroxisome proliferator-activated receptor gamma coactivator 1 (PGC-1) family as a core regulatory component [85]. Among these, PGC-1α is regarded as the principal regulator of mitochondrial biogenesis [86]. It stimulates mitochondrial biogenesis by upregulating nuclear respiratory factors (NRFs) and mitochondrial transcription factor A (TFAM), thereby enhancing mtDNA replication and gene transcription, which ultimately increases mitochondrial number and functional capacity [85].

### 3.2 PQQ and its anti-aging effects

**Fig. 4:**
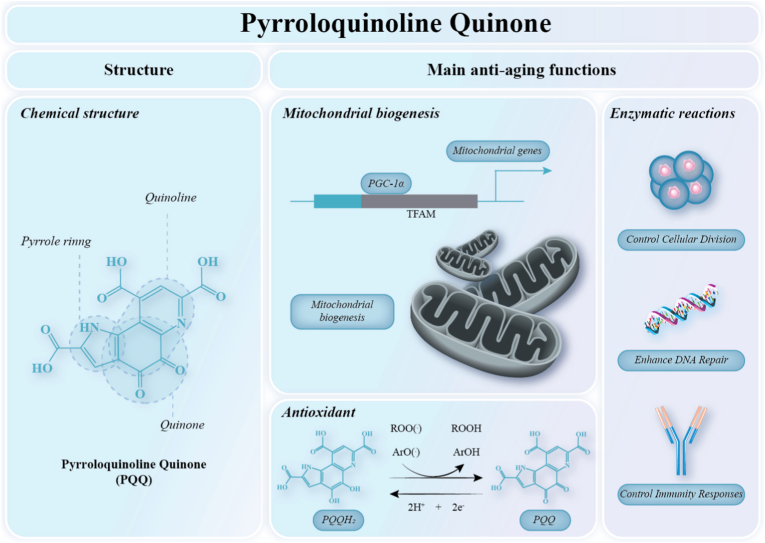
**The Chemical structure and main anti-aging functions of PQQ** ROO(•): Peroxyl radicals; ROOH: Organic hydroperoxide; ArO(•): Aroxyl radicals; ArOH: phenolic antioxidants; PQQH_2_: Pyrroloquinoline quinone dihydroquinone.

Methoxatin or pyrroloquinoline quinone (PQQ) is a redox cofactor and antioxidant that has attracted increasing attention due to its ability to enhance mitochondrial biogenesis and confer multiple health benefits. The structural formula of PQQ is shown in Fig. 4. Pyrroloquinoline quinone has a molecular weight of 330.21 g/mol and functions as a water-soluble vitamin-like compound, a cofactor, an antioxidant, and an anti-inflammatory agent [87]. It is present in a variety of foods, including fermented products, vegetables, and human breast milk [88]. In humans, PQQ is primarily obtained through dietary intake, with a small amount produced by gut microbiota through fermentation. In 1979, PQQ was isolated from bacterial cultures in the form of an acetone adduct, and in 1981, it was chemically synthesized by Corey and Tramontano [89].

Accumulating evidence indicates that PQQ has potential therapeutic relevance in age-related conditions, including neurodegenerative diseases [90], cardiovascular diseases [91], skeletal muscle dysfunction[92], and diabetes mellitus [93]. More importantly, by supporting mitochondrial biogenesis, PQQ increases the number and functional capacity of newly formed mitochondria, enhances cellular energy metabolism, and concurrently protects mitochondria from oxidative stress-mediated lipid peroxidation, protein carbonylation, and respiratory chain damage [94].

The pleiotropic cellular activities of PQQ not only enhance mitochondrial function but also position it as a promising candidate for delaying aging and treating age-related diseases (Fig. 4).

Current experimental evidence suggests that PQQ primarily exerts its biological effects by regulating mitochondrial biogenesis pathways. PQQ has been reported to activate AMPK. When cellular energy is insufficient, an increased AMP to ATP ratio activates AMPK, and PQQ can directly or indirectly promote AMPK phosphorylation and activation [95]. Activated AMPK subsequently phosphorylates PGC-1α, enhancing mitochondrial gene expression and functional maintenance. PQQ enhances the expression of SIRT1 and SIRT3 at both transcriptional and translational levels by targeting PGC-1α, NRF1, NRF2, and TFAM [96]. Studies have demonstrated that PQQ regulates mitochondrial function through the SIRT1 PGC-1α pathway in multiple cell types, including HEI-OC1 [24], NIH/3T3 [97], HepG2 [98,99]. Dietary PQQ supplementation improves mitochondrial content and lipid metabolism in rats [100], and increases mitochondrial number and function in mice, leading to an improved respiratory quotient [101]. Similarly, PQQ supplementation in humans increases peak oxygen consumption and promotes mitochondrial biogenesis by elevating PGC-1α protein levels [102].

Although most studies on PQQ focus on enhancing cellular energy production by improving mitochondrial biogenesis to counteract aging, additional evidence supports its involvement in aging regulation through other mechanisms. For example, as a potent antioxidant, PQQ can neutralize harmful free radicals and reduce excessive oxidative stress and prevent free radical-mediated cellular damage [103]. PQQ is an oxygen-containing quinone with redox activity that can be reversibly reduced to pyrroloquinoline quinone dihydroquinone (PQQH_2_) via a semiquinone intermediate [104]. The reduced PQQH_2_ can then reduce two equivalent molecules of O_2_ to O_2_^−^, thereby oxidizing back to its original quinone state, as shown in Fig. 4. Additionally, PQQ acts as a biocatalyst by promoting NADH production through pyrroloquinoline quinone synthase C. It plays a crucial role in multiple vital processes in bacteria, including m-ATPase-mediated energy transduction, biosynthesis of biocontrol substances, growth promotion, and DNA damage repair [105]. PQQ exhibits anti-inflammatory properties and promotes regenerative potential in aging neuronal cells [106].

### 3.3 Preclinical and emerging clinical evidence

Current studies consistently demonstrate that PQQ regulates mitochondrial biogenesis, energy metabolism, and oxidative homeostasis across cellular, animal, and early clinical settings. The model evidence used in PQQ research is summarized in Table 2.

**Table 2:** **Experimental model evidence for PQQ** IP: Intraperitoneal injection; PO: Per os (oral administration); IG: Intragastric administration.

Study model	Gender	Age/Weight	Route	Dose	Major findings	Reference
Hepa1-6			Add to the culture medium	30 μM for 24h	NRF1/2↑; TFAM, TFB1M and TFB2M↑; PGC-1α↑; Mitochondrial biogenesis↑	[107]
NIH/3T3			Add to the culture medium	10-100 nM for 48h	SIRT1/PGC-1α↑; Mitochondrial biogenesis↑	[97]
HEI-OC1			Add to the culture medium	1.0 nM for 24h	SIRT1/PGC-1α↑; Mitochondrial biogenesis↑	[24]
HepG2			Add to the culture medium	10 and 30 μM for 18, 24, and 48 h	SIRT1/SIRT3↑; PGC-1α↑; NRF1and NRF2↑; Mitochondrial biogenesis↑	[98,99]
C57BL/6	Male	8-week-old and 10-week-old (20-25 g)	PO-water	5, 10, and 20 mg/kg for 14 days	MOTS-c-NRF2↑; Mitochondrial function↑	[108]
ICR	Male	(25-30 g)	IP	20 mg/kg per day for 3 weeks	AMPK↑; Mitochondrial biogenesis↑	[90]
Sprague Dawley	Male	6-week-old (190-220 g)	IG	0.4, 2 and 10 mg/kg for 12 weeks	PGC-1α↑; TFAM↑; Mitochondrial function↑	[109]
C57BL/6J	Male	8-week-old; 83-week-old	PO-diet	20 mg/kg per day for 10 weeks	Mitochondrial function↑	[103]
C57BL/6	Male	8-week-old (28-30 g)	PO-water	4 mg/kg per day for 7 days	PQQ prevents noise-induced and age-related hearing loss	[110]
Healthy adult human beings	Man and Woman	40-year-old to 80-year-old	PO	21.5 mg per day for 12 weeks	Supplementation of PQQ disodium salt is useful in improving memory, attention, judgment, and cognitive function	[111]
Elderly individuals with mild cognitive impairment	Man and Woman	Older than 65-year-old	PO	20 mg per day for 6 weeks	Supplementation of PQQ appears to mildly mitigate cognitive decline	[112]

Substantial research consistently demonstrates that PQQ enhances cellular metabolic adaptability through multiple signaling pathways closely associated with mitochondrial function. Early studies in Hepa1-6 cells confirmed that PQQ significantly stimulates mitochondrial biogenesis by promoting CREB phosphorylation and upregulating PGC-1α expression, activating nuclear respiratory factors NRF-1 and NRF-2, and increasing the transcriptional levels of TFAM, TFB1M, and TFB2M. This process also enhances cellular tolerance to multiple mitochondrial toxicity inhibitors [107]. In NIH/3T3 fibroblasts, PQQ was further demonstrated to be a key mechanism underlying its induction of mitochondrial biogenesis by elevating intracellular NAD^+^ levels and activating the SIRT1/PGC-1α signaling pathway [97]. In aging- or stress-related cellular models, PQQ's mitochondrial-protective effects are particularly pronounced. For instance, in the premature aging model of HEI-OC1 auditory cells, PQQ improves mitochondrial structure and function by restoring SIRT1 expression and the deacetylation state of PGC-1α. This enhances ATP production rates and maximal respiratory capacity, thereby mitigating oxidative stress-induced premature cellular aging [24]. In HepG2 cells, PQQ synchronously upregulates the gene and protein expression of Sirt1 and Sirt3 while enhancing their activity. Their downstream targets, including PGC-1α, NRF-1/2, and TFAM, further strengthen the mitochondrial biogenesis network [98,99]. Overall, cellular-level evidence indicates that PQQ synergistically regulates mitochondrial number, function, and oxidative stress status at multiple nodes through core pathways such as SIRT1/3-PGC-1α-Nrf2, providing a robust molecular basis for its anti-aging potential.

In vivo models have extensively investigated PQQ's effects across diverse scenarios, including natural aging, metabolic disorders, neurodegenerative diseases, and multi-organ dysfunction, further validating the extensibility of its cellular-level actions throughout the entire physiological system. In d-galactose-induced aging models, PQQ improves mitochondrial function, mitigates muscle atrophy and inflammatory responses, and delays the overall aging process [103]. PQQ also exhibits significant mitochondrial protective effects in multiple organ injury models. In radiation-induced lung injury models, PQQ maintains mitochondrial homeostasis through a MOTS-c-dependent mechanism, thereby alleviating inflammation, oxidative stress, and apoptosis [108]. In the cardiovascular system, PQQ pretreatment improves echocardiographic findings induced by pressure overload or angiotensin II by restoring PGC-1α and TFAM expression and enhancing mitochondrial morphology, thereby preventing the onset of chronic heart failure [109]. In models of the nervous and sensory systems, PQQ promotes mitochondrial biogenesis by activating the AMPK pathway, thereby improving mitochondrial dysfunction in rotenone-induced Parkinson's disease models [90]. Additionally, PQQ also exerts protective effects against noise-induced and age-related hearing loss in mice [110]. Overall, in vivo studies support PQQ's potential as a regulator of mitochondrial function and oxidative homeostasis, delaying aging across multiple organs and pathological contexts.

Compared to cellular and animal studies, clinical evidence for PQQ is still in its early stages, yet it has shown positive signals regarding safety and functional improvements. In healthy volunteers, randomized double-blind controlled studies indicate that supplementation with PQQ disodium salt can enhance memory, attention, and judgment, particularly showing a certain alleviating effect on cognitive decline in middle-aged and elderly populations [111]. In elderly individuals with mild cognitive impairment, a randomized controlled trial of short-term (6-week) supplementation with dihydro-PQQ demonstrated that this intervention improved brain metabolic status and moderately enhanced mental orientation abilities. This suggests the potential application value of PQQ as a dietary intervention in cognitive aging [112]. Overall, existing clinical studies support the feasibility and safety of PQQ in improving cognitive function and mitochondrial-related metabolic indicators. However, its long-term anti-aging effects and underlying mechanisms require validation through larger-scale, long-term follow-up clinical trials.

### 3.4 Limitations and cross-axis interaction

Although extensive cellular and animal studies demonstrate that PQQ significantly promotes mitochondrial biogenesis and improves mitochondrial quantity and energy metabolic capacity through activation of PGC-1α, NRF1, NRF2, and TFAM, fine regulation of mitochondrial function depends not only on biogenesis itself but also on multiple additional factors, including NAD^+^ dependent deacetylation, respiratory chain complex assembly efficiency, and proteostasis regulation [113]. Previous studies have shown that during aging, intracellular NAD^+^ depletion and restricted SIRT activity are common biological features [114], which may impose constraints on mitochondrial functional maturation. Moreover, an increase in mitochondrial number does not necessarily lead to a concurrent improvement in redox homeostasis [58,[79], [80], [81]]. Although PQQ possesses strong antioxidant capacity and participates in ROS clearance through repeated redox cycling, some studies have reported that PQQ can induce low-level ROS production at the plasma membrane to promote lifespan extension [115].

In summary, the role of PQQ in anti-aging interventions is more appropriately defined as a representative enhancer of mitochondrial remodeling and quality control, rather than a standalone intervention capable of independently restoring energy metabolism and redox stability.

## 4 Redox stability in aging and ergothioneine

### 4.1 Redox stability in aging

As aging progresses, random mutations and oxidative damage accumulate in mtDNA [116], thereby impairing the structural integrity and electron transport system efficiency and further exacerbating mitochondrial dysfunction. ROS are highly oxidative molecules, including radical species such as superoxide anion radical (O_2_·^−^) and hydroxyl radical (·OH), as well as nonradical oxidants such as hydrogen peroxide (H_2_O_2_) and singlet oxygen (^1^O_2_) [117]. ROS are primarily generated during OXPHOS as a result of electron leakage from mitochondrial electron transport chain complexes I and III [118]. Within a normal physiological range, ROS are not merely harmful byproducts; they actively participate in multiple cellular signaling and adaptive regulatory processes, playing a crucial role in maintaining cellular homeostasis, while reactive nitrogen species (RNS) such as peroxynitrite (ONOO^−^) contribute to oxidative and nitrosative stress. The mitochondrial free radical theory of aging (MFRTA) [119] proposed that aging is driven by mitochondrial ROS-mediated damage to macromolecules. However, recent studies suggest that increased ROS levels may not be the initiating or decisive cause of aging[120], although their levels are closely associated with the aging process.

Under normal physiological conditions, intracellular ROS scavenging systems are sufficient to maintain redox homeostasis. However, aging-related mitochondrial dysfunction reduces electron transfer efficiency and increases electron leakage, leading to ROS production that exceeds cellular clearance capacity and induces oxidative stress [121]. This, in turn, causes further damage to proteins, DNA, and lipids, forming a vicious cycle. Therefore, effectively limiting the excessive accumulation of harmful ROS while preserving their physiological signaling functions has become a key objective in anti-aging interventions. In one study, ergothioneine was shown to exhibit selective antioxidant properties, primarily scavenging the most reactive and destructive hydroxyl radicals, without excessively eliminating physiological ROS required for normal cellular function [122]. Moreover, compared to classic antioxidants such as glutathione, uric acid, and trolox, EGT is the most effective free radical scavenger [123].

### 4.2 Ergothioneine and its anti-aging effects

**Fig. 5:**
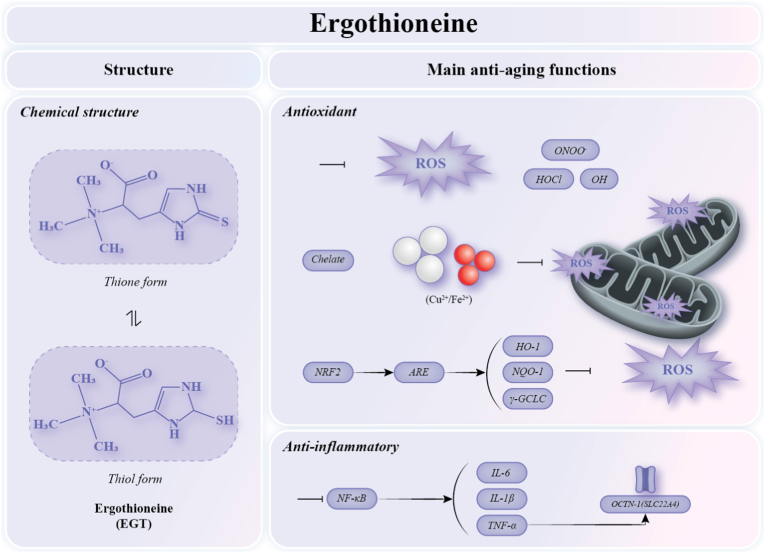
**The Chemical structure and main anti-aging functions of EGT** Ergothioneine exists as a tautomer with both thione and thiol forms, and predominantly exists in the thione tautomer; ROS: Reactive oxygen species; ONOO^−^: Peroxynitrite; HOCl: Hypochlorous acid; ·OH: Hydroxyl radical; NRF2: Nuclear factor erythroid 2-related factor 2; ARE: Antioxidant response element; NF-κB: Nuclear factor κB; IL-6: Interleukin-6; IL-1β: Interleukin-1β; TNF-α: Tumor necrosis factor-α; HO-1: Heme oxygenase 1; NQO-1: NAD(P)H quinone oxidoreductase 1; γ-GCLC: γ-glutamylcysteine ligase; OCTN1: Organic cation transporter novel type 1.

Ergothioneine (EGT) is an amino acid betaine belonging to l-histidine derivatives and is also a sulfur-containing amino acid. The structural formula is shown in Fig. 5, and thiol and thione forms exist as a tautomer. The thione form is more common at physiological pH (around 7.0) and changes into a small amount of the thiol form. This structural characteristic confers stable, highly effective antioxidant activity [124]. Its molecular weight is 229.30 g/mol [125], and it exhibits antioxidant activity as an efficient scavenger of reactive oxygen species. EGT was first isolated in 1909 during studies of the ergot fungus, from which it derives its name [126]. This naturally occurring hydrophilic amino acid is widely distributed in most cells and tissues of plants and mammals. Higher animals cannot synthesize EGT endogenously and must obtain it entirely from the diet, with particularly high concentrations found in mushrooms. Due to its high redox potential and selective reactivity with certain thiol groups [127], EGT exhibits stronger resistance to auto oxidation and more effective free radical scavenging activity compared with conventional antioxidants such as glutathione and vitamin E. Dietary intake of EGT in elderly populations is associated with a significantly reduced risk of cognitive decline and Alzheimer's disease [128], as well as improvements in chronic inflammation [129], cardiovascular disease [130] and renal dysfunction [131].

EGT is one of the few identified nutritional factors capable of entering mitochondria and exerting antioxidant effects within the organelle. In humans, the organic cation transporter novel type 1 (OCTN1), also known as solute carrier family 22 member 4 (SLC22A4), mediates the transport of EGT from the bloodstream into tissues and cells [132]. Through OCTN1-dependent transport, EGT accumulates intracellularly, particularly in mitochondria and the plasma membrane, where it helps mitochondria cope with metabolic stress. Studies have shown that cells lacking OCTN1 are more susceptible to mtDNA damage, protein oxidation, and increased lipid peroxidation [26], although the precise intramitochondrial localization of EGT remains controversial.

The primary anti-aging functions of EGT are illustrated in Fig. 5. It primarily eliminates ROS directly or indirectly through multiple mechanisms, while also exhibiting anti-inflammatory effects.

EGT can directly scavenge or neutralize ROS, RNS, and free radical-derived products, including singlet oxygen (O_2_), hypochlorous acid (HOCl), superoxide (O_2_·^−^), hydroxyl radical (·OH), and peroxynitrite (ONOO^−^)[25]. In addition, EGT prevents ROS formation by forming stable chelate complexes with metal ions [133]. Metal ions, including iron, zinc, and copper, serve as essential cofactors for numerous enzymes and proteins. However, both excess and deficiency of these metal ions can lead to cellular dysfunction. EGT can chelate metal cations such as Cu ^2+^/Cu + in the nucleus and other cellular compartments [134], thereby preventing the Fenton reaction [132] and indirectly reducing the generation of additional free radicals. EGT exerts antioxidant and anti-aging effects through the NRF2 signaling pathway, primarily by enhancing antioxidant enzyme systems such as heme oxygenase 1 (HO-1), NAD(P)H quinone oxidoreductase 1 (NQO-1), and γ-glutamylcysteine ligase (γ-GCLC), thereby reducing ROS production [135]. This mechanism has been demonstrated in UVA induced skin aging models [136] and has also been shown to improve memory impairment in d-galactose induced aging mice [137]. The activity of sirtuin family enzymes can also be modulated by EGT, indirectly affecting NAD transport and cycling between the cytoplasm and mitochondria and influencing mitochondrial ROS levels. EGT has been shown to interfere with hyperglycemia related endothelial cell senescence by regulating SIRT1 and SIRT6 signaling [138]. Collectively, these findings support the role of EGT as an antioxidant and a potential anti-aging agent [139].

Researchers have also discovered that EGT possesses anti-inflammatory effects dependent on the NF-κB pathway. Salama et al. demonstrated that EGT reduces the nuclear translocation of NF-κB p65, thereby decreasing levels of TNF-α, IL-6, and IL-1β [140]. On the other hand, during inflammation, TNF-α has been shown to induce OCTN1 expression via NF-κB [141], thereby enhancing cellular uptake of ergothioneine.

### 4.3 Preclinical and emerging clinical evidence

Evidence from cellular, animal, and preliminary clinical studies suggests that EGT functions as a potent regulator of oxidative homeostasis and mitochondrial protection across multiple biological systems. Its biological effects are primarily mediated through coordinated modulation of antioxidant signaling pathways, including Nrf2-dependent defense mechanisms, sirtuin-mediated metabolic regulation, and maintenance of mitochondrial function. To provide a clearer overview of the experimental systems and physiological contexts in which EGT has been investigated, the representative cellular models, in vivo models, and clinical studies are summarized in Table 3.

**Table 3:** **Experimental model evidence for EGT** IP: Intraperitoneal injection; PO: Per os (oral administration); IG: Intragastric administration; ID: Intradermal injection.

Study model	Gender	Age/Weight	Route	Dose	Major findings	Reference
HT22			Add to the culture medium	100 μg/mL for 8 h	NRF2↑; Antioxidant↑; Mitochondrial function↑	[142]
HaCaT; Human epidermal fibroblasts from Cascade Biologics			Add to the culture medium	0.1, 1, and 10 mM for 2 h.	NRF/HO-1↑; HSP70↑; ROS↓; PARP, Caspase-8 ↓; IL-1β, IL-6, TNFα↓	[143]
HSF			Add to the culture medium	0.125-0.5 μM for 24 h	ROS↓; HO-1, NQO-1, GCLC↑; AP-1↓; NRF2↑	[136]
HT22			Add to the culture medium	140 μM for 6, 8, 12, and 24 h	ROS↓	[144]
C57BL/6J	Male	6-week-old (20-21 g)	IG	10, 20, and 40 mg/kg for 11 weeks	NRF2↑; HO-1↑; MDA↓; Mitochondrial function↑	[137]
C57BL/6	Male	8-week-old	PO-diet	5.6 ng/mg for 2 weeks	3-mercaptopyruvate sulfurtransferase ↑; Mitochondrial function↑	[145]
C57BL/6	Male	4-week-old; 30-month-old	ID	70 mg/kg every other day for 14 days	SIRT1/NRF2↑; ROS↓; IL-1β, IL-6, TNFα↓; SCF, SDF1↑	[57,58]
C57BL/6	Male	10-week-old	IG	40 mg/kg per day for 2 months	SIRT6↑; NF-κB↓; IL-1β↓	[146]
C57BL/6 N	Male	8-week-old	PO-diet	70 mg/kg per day for 12 months	ROS↓; NRF2↑; HO-1↑;	[147]
Sprague Dawley	Male	(175 ± 20 g)	PO-water	35 mg/kg for 7 weeks	HO-1, NQO-1↑; AP-1↓; NRF2↑; NF-κB↓; TGF-β1↓	[131]
Elderly individuals with mild cognitive impairment	Male and Female	60-year-old to 90-year-old	PO	25 mg, third a week for 1 year	Delay cognitive decline in elderly adults	[148]

At the cellular level, EGT is primarily used to investigate its roles in oxidative stress defense, mitochondrial function maintenance, regulation of cellular senescence phenotypes, and suppression of inflammatory paracrine signaling. EGT-rich shiitake mushroom extract significantly restores mitochondrial function in aged HT22 cells. This effect is closely associated with the activation of the Nrf2 signaling pathway and the upregulation of genes related to mitochondrial biogenesis, suggesting that EGT exerts direct mitochondrial protective effects during neuronal aging [142]. Similarly, mushroom preparations rich in EGT have been demonstrated to act as potential neuroprotective anti-aging candidates by scavenging free radicals and reducing the accumulation of senescent cells [144]. The anti-photoaging effects of EGT exhibit highly consistent mechanisms, namely by inhibiting ROS production and maintaining the activity of antioxidant defense pathways to delay cellular aging processes. In UVB [143] or UVA [136]-induced injury models, EGT prevents the downregulation of the Nrf2/HO-1 pathway, HSP70, and multiple Nrf2 target genes while inhibiting AP-1 signaling and pro-apoptotic protein activation, thereby reducing oxidative damage and maintaining collagen homeostasis. In metabolic stress-related vascular endothelial cell models, EGT's inhibitory effect on cellular senescence further demonstrates the synergistic regulation of antioxidant and anti-inflammatory signaling. During high-glucose-induced endothelial senescence, EGT significantly reduces ROS levels and cytotoxicity, alleviates inflammatory responses, and delays cellular senescence by upregulating SIRT1 and SIRT6 while inhibiting p66Shc and NF-κB signaling [138]. Overall, cellular evidence indicates that EGT exerts comprehensive protective effects against oxidation, inflammation, and aging across multiple cell types, primarily through the Nrf2-SIRT-mitochondrial homeostasis axis.

In living models, EGT's anti-aging effects extend from cellular-level regulation of oxidative homeostasis to the maintenance of multi-organ function and overall delay of the aging process. In neurological aging models, EGT significantly improves learning and memory abilities in d-galactose-induced mouse models. This effect is closely associated with Nrf2/HO-1 activation, reduced oxidative stress, and AMPK/SIRT1/PGC-1α-mediated improvements in mitochondrial function [137]. In the fly model, EGT extends lifespan and improves overall health by regulating neurotransmitter metabolism, fatty acid oxidation, and autophagy, further supporting its fundamental role in neuroaging [149]. At the level of exercise and energy metabolism, EGT enhances mitochondrial respiratory function by directly binding to and activating 3-mercaptopyrate sulfurtransferase (MPST), thereby significantly improving exercise performance and endurance in mice. This suggests its potential value in maintaining energy metabolism functions associated with aging [145]. Regarding skin and appendage aging, EGT mitigates UV-induced oxidative damage, inhibits collagen degradation, and improves the follicular microenvironment through the PI3K/Akt/Nrf2 and SIRT1/Nrf2 pathways, thereby slowing down the processes of skin photoaging and follicular aging [57,58,150,151]. Furthermore, in models of degenerative bone and joint diseases and metabolic organ aging, EGT alleviates inflammatory responses and inhibits osteoarthritis progression, both in vivo and in vitro, by activating SIRT6 and suppressing NF-κB [146]. In cardiovascular [147], and renal [131] aging models, EGT primarily enhances organ function and delays damage progression through Nrf2-mediated antioxidant defense enhancement, inflammation suppression, and tissue-specific accumulation (e.g., OCTN1-mediated hepatic enrichment). Collectively, in vivo studies support EGT as a cross-organ regulator of oxidative homeostasis and mitochondrial function, providing fundamental protective effects during multisystem aging.

Compared to cell and animal studies, clinical research on EGT remains in its exploratory phase, yet it has preliminarily demonstrated safety and potential value in cognitive aging intervention. A preliminary study involving subjects aged 60 and above with mild cognitive impairment indicated that long-term EGT intake exhibits a favorable safety profile with no observed toxic reactions, suggesting its potential in slowing cognitive decline [148]. These findings provide preliminary clinical evidence supporting further investigation of EGT as a dietary or nutritional intervention for maintaining age-related cognitive function.

### 4.4 Redox stabilization as a permissive factor

EGT primarily contributes to mitochondrial homeostasis by buffering excessive oxidative stress and preserving mitochondrial integrity under conditions of increased metabolic and remodeling demand. However, redox buffering alone is unlikely to fully restore mitochondrial abundance or bioenergetic performance in aging. In contrast, NMN/NR replenishes NAD^+^ precursors and activates deacetylases such as SIRT1 and SIRT3, thereby optimizing mitochondrial protein function and respiratory chain activity. PQQ, meanwhile, promotes mitochondrial biogenesis by activating the PGC-1α and AMPK signaling pathways, leading to increased mitochondrial number and overall metabolic capacity.

Under conditions of enhanced energy metabolism, increased respiratory chain flux may elevate ROS levels. The selective buffering capacity of EGT constrains ROS-mediated damage to both newly generated and pre-existing mitochondria, thereby preserving system-level homeostasis. Within the present framework, NMN/NR provides broad NAD^+^-dependent metabolic and signaling support, whereas PQQ and EGT may further enhance mitochondrial adaptation by strengthening remodeling capacity and limiting ROS-associated damage, respectively. Together, this integrated strategy enables coordinated improvements in mitochondrial quantity, function, and stability in the context of aging.

## 5 Integrated tri-axis anti-aging model

### 5.1 Conceptual framework

Age-related mitochondrial dysfunction does not arise from a single molecular lesion, but from a progressive imbalance among metabolic regulation, mitochondrial remodeling, and redox control. These processes are highly interconnected within cells and cannot be strictly separated mechanistically. Declining NAD^+^ availability, impaired mitochondrial renewal, and reduced redox resilience together constrain mitochondrial adaptability during aging, making single-pathway interventions unlikely to fully restore mitochondrial homeostasis.Based on the systematic integration of prior mechanistic evidence, this paper concurs a comprehensive anti-aging framework, presented in Table 4, centered on NMN/NR, PQQ, and EGT. This framework does not define the three as functionally isolated interventions but emphasizes a synergistic relationship characterized by “dominant function + limited overlap”, reflecting the highly coupled nature of mitochondrial regulatory networks. Within this model, NMN/NR acts as a broad NAD^+^-dependent regulator of mitochondrial homeostasis. Its most immediate and best-established effects are metabolic, including support of glycolysis, fatty acid oxidation, the tricarboxylic acid cycle, and oxidative phosphorylation. However, the biological consequences of NAD^+^ restoration extend beyond energy metabolism alone. Through sirtuin-dependent pathways and related signaling networks, NMN/NR may also influence mitochondrial quality control, mitophagy-related processes, stress adaptation, and biogenesis-associated signaling. In this context, PQQ is incorporated not because mitochondrial remodeling is absent from NMN/NR biology, but because PQQ may further strengthen this dimension under conditions in which mitochondrial renewal becomes constrained during aging. By promoting mitochondrial biogenesis, adaptive remodeling, and aspects of mitochondrial quality control, PQQ may help expand the functional mitochondrial pool and support longer-term structural and metabolic adaptation. Similarly, EGT is incorporated because EGT may further reinforce redox buffering capacity under conditions of increased metabolic and remodeling demand. By limiting excessive ROS-associated damage and preserving mitochondrial integrity, EGT may help sustain the cellular environment required for ongoing mitochondrial adaptation.

**Table 4:** Predominant functional contributions of NMN/NR, PQQ, and EGT within mitochondrial homeostasis.

Category	NMN/NR	PQQ	EGT
**Chemical nature**	NAD^+^ precursor	Redox-active quinone	Sulfur-containing thiol antioxidant
**Molecular weight**	NMN ≈334 g/mol; NR ≈ 255.25 g/mol	≈330 g/mol	≈229 g/mol
**Biological origin**	Endogenous synthesis; dietary sources	Microbial origin; trace dietary intake	Diet-derived (synthesized by fungi/bacteria)
**Primary cellular localization**	Cytosol and nucleus	Cytosol and mitochondria-associated compartments	Mitochondria-enriched (via OCTN1 transport)
**Mode of mitochondrial action**	Indirect (via NAD^+^-dependent nuclear-mitochondrial signaling)	Semi-direct (signaling-mediated biogenesis & structural effects)	Direct mitochondrial protection
**Key signaling pathways**	NAD^+^ → Sirtuin; mitophagy- and stress-adaptation-related pathways	PGC-1α-NRF-TFAM-mediated mitochondrial biogenesis	OCTN1-mediated uptake; redox buffering
**Impact on mitochondrial homeostasis**	Supports NAD^+^-dependent quality control, mitophagy, and redox-associated stress responses	Enhances biogenesis and structural integrity of mitochondrial networks	Preserves redox balance and protects membrane integrity
**Functional dimension targeted**	NAD^+^-dependent metabolic activation with broad downstream effects	Mitochondrial remodeling and quality control enhancement	Redox buffering and stress resistance

Therefore, the rationale of this framework is not exclusivity, but reinforcement. Through synergistic regulation of NAD^+^-dependent metabolic support, mitochondrial remodeling, and redox buffering, they achieve sustainable restoration of mitochondrial homeostasis under senescent conditions.

### 5.2 Mechanistic interactions

**Fig. 6:**
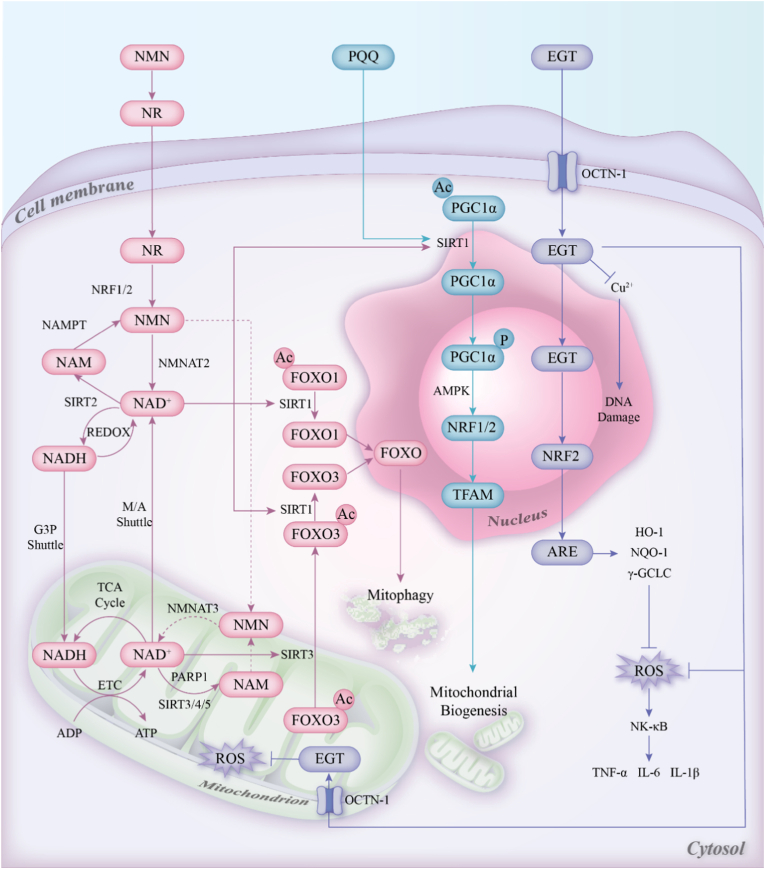
**Potential common mechanism of action for NMN/NR, PQQ, and EGT** Ac: Acetylation; ADP: Adenosine diphosphate; AMPK: AMP-activated protein kinase; ARE: Antioxidant response element; ATP: Adenosine triphosphate; ETC: Electron transport chain; FOXO1/3: Forkhead box O transcription factors 1/3; G3P shuttle: Glycerol-3-phosphate shuttle; HO-1: Heme oxygenase 1; IL-1β: Interleukin-1β; IL-6: Interleukin-6; M/A shuttle: Malate-aspartate shuttle; NAD^+^: Nicotinamide adenine dinucleotide (oxidized); NADH: Nicotinamide adenine dinucleotide (reduced); NF-κB: Nuclear factor κB; NQO-1: NAD(P)H quinone oxidoreductase 1; NR: Nicotinamide riboside; NRF1, 2: Nuclear respiratory factor 1,2; OCTN-1: Organic cation transporter 1 (SLC22A4); P: Phosphorylation; PARP1: Poly(ADP-ribose) polymerase 1; PGC-1α: Peroxisome proliferator-activated receptor gamma coactivator 1-α; ROS: Reactive oxygen species; SIRT1,2,3,4,5: Sirtuin 1,2,3,4,5; TCA cycle: Tricarboxylic acid cycle; TFAM: Mitochondrial transcription factor A; TNF-α: Tumor necrosis factor-α; γ-GCLC: γ-glutamylcysteine ligase.

At the molecular mechanism level, this tri-axial framework reflects a set of partially overlapping and mutually reinforcing processes rather than three isolated pathways. Mitochondrial metabolism, remodeling, and redox regulation are tightly coupled, such that perturbation in one dimension can progressively destabilize the others during aging. The key mechanisms of these three molecules within cells are illustrated in Fig. 6.

NMN/NR acts at the core of this network by restoring intracellular NAD^+^ availability. Increased NAD^+^ supports metabolic flux through glycolysis, fatty acid oxidation, the TCA cycle, and oxidative phosphorylation, while also reactivating NAD^+^-dependent signaling pathways, particularly sirtuins. Through these mechanisms, NMN/NR may influence not only mitochondrial bioenergetics but also stress adaptation, mitophagy-related regulation, and broader mitochondrial quality control. PQQ contributes mainly by enhancing mitochondrial biogenesis, renewal, and adaptive remodeling. By activating the PGC-1α signaling axis, PQQ promotes mitochondrial biogenesis and accelerates the replacement of dysfunctional mitochondria. Newly generated mitochondria typically exhibit superior membrane integrity, respiratory chain complex assembly, and redox efficiency compared to aged mitochondria. However, enhanced metabolism coupled with mitochondrial network expansion increases the system's dependence on redox regulation. Under conditions of elevated metabolic flux and insufficient buffering capacity, adaptive remodeling may transition into oxidative damage. Ergothioneine exerts its stabilizing effect precisely at this level: it accumulates within mitochondria via the OCTN1 transporter, selectively scavenging highly reactive ROS and reactive nitrogen species, and maintains mitochondrial membrane structure, mtDNA stability, and protein functional integrity by activating the Nrf2-dependent antioxidant transcriptional program. Beyond direct redox buffering, ergothioneine indirectly conserves NAD^+^ by limiting ROS-induced PARP overactivation, thereby supporting sustained NAD^+^ levels and sirtuin activity. This feedback mechanism demonstrates that redox stability not only safeguards mitochondrial structure but also critically underpins the ongoing availability of metabolic signaling pathways.

Collectively, NMN/NR, PQQ, and EGT should not be viewed as acting on isolated tiers, but as partially overlapping modulators within a coupled system. NMN/NR broadly restores NAD^+^-dependent metabolic and signaling permissiveness, whereas PQQ and EGT may further reinforce mitochondrial remodeling and redox resilience, respectively, thereby stabilizing mitochondrial adaptation under aging-associated stress. Together, they form a closed-loop mechanism providing a logically coherent and mechanistically clear theoretical framework for multi-target anti-aging interventions.

### 5.3 The possibility and evidence of combination

In current scientific research, studies on NMN/NR, PQQ, and EGT have primarily focused on the biological effects of individual components or pairs of combinations, with very limited systematic elucidation of the molecular mechanisms underlying their synergistic interactions. However, at the application and translation level, a few relevant patents have pioneered explorations of multi-component combination strategies, providing preliminary yet crucial evidence supporting multi-target anti-aging intervention models. Particularly in the fields of anti-aging and mitochondrial function regulation, multiple commercial or near-commercial formulations have attempted to combine NMN/NR, PQQ, and EGT at varying levels, reflecting sustained industry interest in multi-pathway synergistic strategies to regulate mitochondrial homeostasis. These evidences provide the practical foundation for the framework suggested in this review.

In the NMN/NR -EGT combination, relevant patents primarily focus on delaying the aging process, improving bodily functional states, and enhancing antioxidant capacity. Such patents typically position NMN as the core active ingredient, with potential metabolic support and NAD^+^ supplementation, while designating EGT as an antioxidant that provides cellular protection and oxidative buffering during long-term application. Certain patents propose NMN -containing biological preparations combined with ingredients like EGT to extend lifespan and improve physiological function, emphasizing the stability and safety of this combination during long-term use [152]. This design approach generally posits that incorporating antioxidant components alongside metabolic support may help mitigate age-related functional decline. The underlying rationale is that introducing stable antioxidant compounds within a metabolic support framework could slow age-related functional deterioration. Another patent explicitly combines NMN with EGT to prepare anti-aging and anti-inflammatory formulations, highlighting the potential value of this combination in reducing inflammatory responses and delaying cellular aging [153].

The combination of PQQ and EGT is frequently used in patents within the fields of anti-aging, antioxidant properties, and functional foods or nutritional supplements. Such patents typically emphasize the synergistic effects of these two compounds in improving bodily condition, reducing oxidative stress, and maintaining functional homeostasis. For instance, one patent proposes nutritional capsules containing PQQ and EGT as core ingredients to improve sleep quality and support overall health, highlighting the combination's advantages in safety and stability for long-term use [154]. The patent further incorporates multiple compounds, including PQQ, EGT, and superoxide dismutase, into a base containing cranberry powder, thereby providing a method for preparing a solid beverage for beauty and anti-aging purposes. It highlights its potential applications in improving bodily condition and delaying signs of aging [155]. Overall, the PQQ-EGT complex primarily functions as a regulator and maintainer of oxidative homeostasis within the existing patent system. Its action axis is predominantly focused on maintaining steady-state conditions rather than directly intervening in NAD-related metabolic processes.

In contrast, the combination of NMN/NR and PQQ appears frequently in patents, widely applied in anti-aging functional formulations, nutritional preparations, and related product designs. Notably, such patents often incorporate additional antioxidant or stabilizing components beyond NMN and PQQ to enhance the practical feasibility and system stability of the combination. Patent evidence suggests that this combination primarily targets improvements in metabolic function and enhanced anti-aging effects. For instance, one patent highlights that betaine effectively reduces the oxidation of NMN, PQQ, and quercetin, thereby further enhancing their synergistic antioxidant, anti-inflammatory, and anti-aging activities when combined [156]. Another patent proposes using a giant salamander oligopeptide to inhibit free radical-induced oxidative stress, thereby enhancing the stability of NMN and PQQ's anti-aging effects within the system [157]. Additionally, patents explicitly specify NMN (0.48-0.53), PQQ (0.012-0.018), coenzyme Q10, ginsenoside (0.113-0.118), aminobutyric acid (0.162-0.167), and celery seed extract (0.16-0.166), emphasizing their synergistic effects in delaying aging [158]. This combination approach has also been extended to the field of pet health. The combination of NMN, PQQ, and CoQ can help increase mitochondrial numbers in pet cells, enhance mitochondrial activity, and effectively elevate NAD^+^ levels, thereby improving mitochondrial functional status [159].

Building on this foundation, a three-component combination of NMN/NR, PQQ, and EGT has been proposed in several patents. These patents typically target core objectives such as delaying aging, reducing inflammation, and maintaining stable bodily functions. They treat the three components as distinct active modules with clearly defined functional roles that complement each other, rather than a simple additive combination of nutrients. One patent explicitly proposes combining NMN/NR, PQQ, and EGT in specific ratios (1-100 parts of ergothioneine, 1-100 parts of PQQ or a derivative thereof, and 20-1000 parts of component C, wherein component C is NMN, NR, or a derivative thereof) for anti-aging and anti-inflammatory applications. These patents emphasize that the synergistic effects of the three components in slowing the aging process surpass those of single components or simple two-component combinations [160]. A patented approach proposes a mature formulation of PQQ, EGT, and NAD in a 1:1:1 ratio for delaying skin aging. This formulation is discussed as supporting NAD^+^ in activating the Sirtuin protein family and repairing DNA damage, while PQQ promotes the production of new mitochondria. EGT primarily assumes antioxidant and cell-protective functions, collectively establishing a multi-tiered anti-aging strategy [161].

Overall, existing patent evidence suggests that industrial and applied sectors have begun exploring multi-component synergistic strategies involving NMN/NR, PQQ, and EGT to simultaneously address key aspects, including metabolic support, enhanced mitochondrial function, and protection against oxidative damage. These explorations partially address the current research gap in understanding the synergistic mechanisms among these three compounds. They also provide crucial clues and practical foundations for systematically integrating the present multi-dimensional framework of NAD^+^-dependent metabolic support, mitochondrial remodeling, and redox buffering.

### 5.4 Additional mitochondria-targeted therapeutics

The present tri-axis model highlights NMN/NR, PQQ, and EGT as representative modulators of mitochondrial metabolism, remodeling, and redox balance. It is important to note that mitochondria-targeted interventions are rapidly expanding. A growing number of compounds have been identified that may exert beneficial effects on mitochondrial homeostasis. Table 5 summarizes their proposed mechanisms of action and distinguishes between preclinical and clinical evidence, where available. Although many of these molecules remain at the preclinical stage, they provide important insights into potential therapeutic directions.

**Table 5:** Emerging mitochondria-targeted compounds and supporting evidence.

Effect on mitochondria	Compound	Mechanism	Preclinical evidence	Clinical evidence	Ref
Reduction of oxidative damage	Coenzyme Q10	Scavenges ROS and reduces lipid peroxidation	Supplementation reduces ROS and reverses age-associated memory decline in mice	Clinical studies suggest reduced myocardial CoQ10 levels are associated with increased severity of heart failure	[162]
	Alpha-lipoic acid	Direct antioxidant effects and regeneration of endogenous antioxidants	Reverses age-related cognitive decline, reduces mtDNA damage, and enhances antioxidant enzyme expression in aged rats	Numerous clinical studies have shown that it exerts a protective effect in biological systems by directly or indirectly quenching reactive oxygen and nitrogen species	[162,163]
Enhancement of mitochondrial proteostasis	Urolithin A	Activates transcriptional programs related to mitochondrial biogenesis and mitophagy (NRF1/NRF2 pathways)	First demonstrated to inhibit Alzheimer's disease progression in animal models	500–1000 mg doses improve skeletal muscle mitophagy and mitochondrial metabolism in humans	[31,32]
	Spermidine	Inhibits acetyltransferase p300, thereby promoting autophagy-related gene expression	Enhances cardiac autophagy and mitophagy, improves mitochondrial respiration in mice	The human group with the highest dietary spermidine intake had a lower mortality rate	[162,164]
	Trehalose	Activates TFEB-mediated autophagy and reduces p62/SQSTM1, promotes LC3 conversion	Improves motor function and extends lifespan in Huntington's disease mouse models	it is safe and well-tolerated, while trehalose is not an effective treatment for slowing the progression of amyotrophic lateral sclerosis in the selected study population at the selected dose.	[162,165]
	Kaempferol	Increases levels of PINK1, Parkin, Beclin-1, LC3B-II, and AMBRA1	Improves neuronal survival and function in AD models via autophagy induction	Daily intake of kaempferol ≥1.5 mg/day was associated with lower CHD mortality and MI incidence	[33,166]
	Rhapontigenin	Increases the phosphorylation level of the autophagy factor ULK1 at Ser555	Enhances neuronal survival and function via autophagy pathways in AD models	-	[33]
	Tomatidine	Activates SKN-1/Nrf2 signaling pathway to induce mitophagy	Extends lifespan and healthspan in *C. elegans* via mitophagy induction	-	[167]
	α-amyrin	Modulates the DLK-SARM1-ULK1	Preserving memory in animal models of AD	-	[168]
	Isoginkgetin	Regulates GSK-3β–TFEB signaling axis	Protects motor neurons in ALS models and promotes autophagy/mitophagy	-	[169]
Regulation of mitochondrial energy metabolism	Metformin	Activates AMPK signaling and enhances metabolic regulation	Binds PEN2 and modulates lysosomal signaling; improves mitochondrial metabolism in animal models	Side effects include gastrointestinal disturbance and rare lactic acidosis	[162]
	Mitochondria-targeted tamoxifen	Reduces oxidative phosphorylation and mitochondrial membrane potential in senescent cells	Reduces aging markers in multiple organs after short-term treatment in aged mice	-	[162]

These compounds can be broadly categorized based on their predominant functional effects on mitochondria, including reduction of oxidative damage, enhancement of mitochondrial proteostasis, and regulation of mitochondrial energy metabolism (Table 5). For example, classical antioxidants such as coenzyme Q10 and α-lipoic acid primarily function by reducing mitochondrial oxidative stress and improving redox balance, thereby indirectly preserving mitochondrial integrity.

In contrast, a growing number of molecules have been identified that more directly influence mitochondrial quality control, particularly through modulation of autophagy and mitophagy pathways. Urolithin A represents one of the most extensively studied compounds in this category, with evidence demonstrating its ability to enhance mitochondrial turnover and improve mitochondrial function in both animal models and early clinical studies. Similarly, spermidine and trehalose have been shown to promote autophagic processes and improve mitochondrial function in preclinical systems, although their clinical efficacy remains limited or context-dependent. Additional natural compounds, including kaempferol and rhapontigenin, further support the concept that targeting mitophagy-related signaling pathways may improve neuronal survival and mitochondrial function, particularly in models of neurodegenerative diseases. More recently, emerging molecules such as tomatidine, α-amyrin, and isoginkgetin have been reported to modulate key regulators of mitochondrial quality control, including Nrf2-, ULK1-, and TFEB-related pathways. Although these findings are still largely confined to experimental systems, they highlight the expanding landscape of mitochondria-targeted therapeutic development.

Beyond quality control, metabolic regulators such as metformin also influence mitochondrial function through activation of AMPK signaling and downstream metabolic pathways. In addition, mitochondria-targeted derivatives of existing drugs, such as mitochondria-targeted tamoxifen, have been explored for their ability to selectively modulate mitochondrial bioenergetics in aging cells.

Collectively, these observations indicate that mitochondria-targeted therapeutics encompass a diverse and rapidly evolving field. Importantly, many of these compounds converge on common regulatory processes, particularly mitochondrial quality control and mitophagy, supporting the central role of these pathways in aging. While further clinical validation is required, these emerging strategies provide a broader context for the tri-axis model and suggest that future anti-aging interventions may benefit from integrating metabolic activation, mitochondrial remodeling, and redox regulation with targeted modulation of mitochondrial turnover.

## 6 Future perspectives and applications

The aging process involves multi-system, multi-level functional decline characterized by mitochondrial dysfunction, NAD^+^ depletion, chronic low-grade inflammation, and cumulative oxidative stress. These alterations disrupt local tissue homeostasis while amplifying effects across systemic metabolic, immune, and endocrine networks. The nervous, skin, metabolic-endocrine, musculoskeletal, immune, cardiovascular, and reproductive systems exhibit high heterogeneity during aging: organ-specific functional losses coexist with shared impairments in energy metabolism and antioxidant defenses. Against this complex backdrop, single-target interventions struggle to sustain long-term homeostasis. A multi-tiered combination approach, boosting NAD^+^ levels with NMN/NR to activate sirtuin signaling and improve energy metabolism and autophagy; enhancing mitochondrial renewal and antioxidant tolerance with PQQ to maintain cellular homeostasis; and eliminating ROS while modulating chronic inflammation with EGT, holds promise for concurrently exerting protective effects across multiple systems. Current evidence is shown in Table 6 to provide both theoretical foundations and experimental support for its potential in delaying systemic aging and related diseases (see Table 7).

**Table 6:** Comparison of the anti-aging efficacy of NMN/NR, PQQ, and EGT in different diseases.

Disease	NMN/NR			PQQ			EGT		
	Function	Mechanism	Ref	Function	Mechanism	Ref	Function	Mechanism	Ref
**Nervous System Aging and Neurodegenerative Disorders**	Restores angiogenic capacity of aged cerebral vascular endothelial cells and prevents vascular cognitive impairment.	Restores intracellular NAD^+^ levels; activates SIRT1-dependent pathways; promotes endothelial proliferation, migration, and angiogenesis.	[69]	Promotes mitochondrial biogenesis in rotenone-induced Parkinson's disease models.	Activates AMPK signaling; enhances mitochondrial biogenesis in rotenone-injured mice and SH-SY5Y cells.	[90]	Eliminates senescent neurons.	Scavenges free radicals; suppresses senescence markers p21^CIP1^ and p16^INK4a^.	[144]
	Alleviates cognitive impairment following chronic cerebral hypoperfusion.	Reduces microglial activation and polarization; inhibits excessive microglial phagocytosis of myelin.	[170]	Reduces glutamate neurotoxicity and improves d-galactose-induced cognitive impairment	Modulates pro-inflammatory mediators including cytokines and prostaglandins	[171]	Improves cognitive function in d-galactose-induced aging models.	Activates Nrf2/HO-1 and AMPK/SIRT1/PGC-1α pathways; reduces oxidative stress and hippocampal neuronal injury.	[137]
	Reverses d-galactose-induced neurodegeneration and improves intestinal barrier integrity.	Activates SIRT1/AMPK/PGC-1α signaling; coordinates antioxidant, anti-inflammatory, and anti-apoptotic responses.	[172]	Improves noise-induced and age-related hearing loss	Preserves inner and outer hair cells (IHCs, OHCs), synaptic ribbons, nerve fibers, stria vascularis, and spiral ganglion cells	[110]	Delays cognitive decline in subjects with mild cognitive impairment.	Improves learning ability; stabilizes plasma neurofilament light chain levels.	[148]
	Promotes neurovascular rejuvenation in aged mice.	Restores NAD^+^ in senescent neurovascular units; activates sirtuin-mediated transcriptional programs; reduces mitochondrial ROS.	[62]	Enhances memory, attention, judgment, and overall cognitive function.		[111]	Alleviates Alzheimer's disease pathology in 5XFAD mice.	Reduces amyloid plaque burden; attenuates oxidative stress; restores glucose metabolism.	[173]
				Improves mental orientation and cerebral metabolic activity in elderly patients with mild cognitive impairment.		[112]			
**Integumentary System Aging (Skin Aging)**	Demonstrates anti-aging effects in human skin fibroblasts.	Elevates intracellular NAD^+^; activates sirtuin signaling and autophagy.	[68]	Protects against UV-induced senescence in human dermal fibroblasts.	Activates anti-apoptotic SIRT1/Nrf2/HO-1 and SIRT6 pathways.	[98,99]	Alleviates UVB-induced aging of fibroblasts.	Protects keratinocytes by preventing the downregulation of the Nrf2/HO-1 pathway and HSP70, while suppressing ROS production and the cleavage of pro-apoptotic proteins, including caspase-8 and PARP; reduces paracrine pro-inflammatory cytokines, including IL-1β, IL-6, and TNF-α.	[143]
	Inhibits d-galactose-induced skin aging in mice.	Activates NAD^+^/SIRT3-mediated mitophagy.	[174]	Delays skin aging in Bmi-1 knockout mice.	Promotes cell proliferation; suppresses p16, p19, and p53; enhances autophagy.	[58,[79], [80], [81]]	Delays the aging of human dermal fibroblasts induced by UVA irradiation.	Attenuates ROS generation and enhances the expression of antioxidant genes (HO-1, NQO-1, and GCLC) through upregulation of Nrf2.	[136]
	Enhances skin barrier function and attenuates UVB-induced photoaging.	Suppresses MAPK phosphorylation; reduces TNF-α and IL-6; downregulates MMP-1; restores HAS-1 and HAS-2 expression.	[175]				Modulates paracrine signaling in dermal papilla cells, counteracts oxidative stress, and inhibits physiological aging of hair follicles.	Attenuates H_2_O_2_-induced damage in dermal papilla cells via the SIRT1/Nrf2 pathway, reduces the secretion of inflammatory cytokines (IL-6, IL-1β, and TNF-α), and enhances the production of pigmentation-promoting factors (SCF and SDF-1).	[57,58]
							Protects against UV-induced oxidative stress.	Reduces malondialdehyde levels and increases SOD activity in UV-irradiated mouse skin; activates the PI3K/Akt/Nrf2 signaling pathway, promotes nuclear translocation of Nrf2, and enhances the expression of Nrf2 target genes.	[150,151]
**Metabolic and Endocrine System Disorders**	Inhibits lung adenocarcinoma growth.	Targets SIRT1 and AMPK/ACC signaling to induce ferroptosis.	[70]	Alleviates oxidative stress and hyperlipidemia in aged rats.	Restores mitochondrial health by stimulating mitochondrial biogenesis and metabolic activity, thereby reducing age-associated oxidative stress and hyperlipidemia.	[176]	Protects endothelial cells from high-glucose-induced senescence.	Upregulates SIRT1 and SIRT6; downregulates p66Shc and NF-κB.	[138]
	Improves glucose intolerance and insulin sensitivity in diet- and age-induced diabetes models.	Restores NAD^+^ levels; activates SIRT1; modulates oxidative stress, inflammation, and circadian rhythm genes.	[53]	Exerts hepatoprotective effects in a high-fat diet-induced MAFLD chicken model.	Enhances mitochondrial biogenesis, thereby increasing antioxidant capacity and anti-apoptotic potential, improving lipid metabolism, and enhancing hepatocellular tolerance to lipid degradation and oxidative injury.	[177]	Exerts beneficial effects on healthspan and lifespan.	Maintains central nervous system homeostasis by coordinating cholinergic neurotransmission, tyrosine metabolism, and peroxisomal protein regulation; modulates autophagic activity via regulation of the lysosomal protease CTSD; preserves mitochondrial function by controlling substrate entry into the tricarboxylic acid cycle.	[149]
				Reduces radiation-induced lung injury.	Maintains mitochondrial function through a MOTS-c-dependent mechanism.	[108]	Improves renal dysfunction in type 2 diabetes models.	Activates Nrf2; induces HO-1 and NQO1; suppresses NF-κB and TGF-β1.	[131]
**Musculoskeletal System Aging**	Increases NAD^+^ levels and improves muscle function in elderly subjects.	Elevates NAD^+^ and NAD metabolites.	[76]	Suppresses age-related intervertebral disc degeneration.	Disrupts Keap1-Nrf2 complex; activates Nrf2-ARE and Wnt5a signaling.	[178]	Improves the phenotype of neonatal puppies and increases survival in mouse models of spinal muscular atrophy.	Stimulates mitophagy, leading to the selective elimination of dysfunctional mitochondria in the diaphragm.	[179]
	Attenuates osteoblast senescence and promotes bone regeneration.	Restores NAD^+^; reverses TNF-α-induced impairment of osteogenic metabolism.	[180]	Delays the progression of aging in male mice and d-galactose-induced cellular senescence, with a pronounced protective effect against muscle atrophy and muscle weakness.	Improves mitochondrial function; reduces oxidative stress and inflammation.	[103]	Modulates specific hematological parameters in Arabian horses.	Regulates post-exercise erythrocyte and leukocyte counts, modulates mean corpuscular volume, adjusts the neutrophil-to-lymphocyte ratio, and stabilizes erythrocyte membrane integrity.	[181]
				Alleviates age-related osteoporosis in naturally aged male mice.	Activates Nrf2 to suppress bone resorption by enhancing cellular stress-response capacity and transcriptionally upregulating Fbn1, thereby reducing RANKL production in osteoblast-lineage cells, limiting osteoclast activation, attenuating osteoblast DNA damage, and delaying osteocyte senescence.	[63,64]	Enhances mitochondrial respiration and exercise performance.	Activates 3-mercaptopyruvate sulfurtransferase.	[145]
				Prevents estrogen-deficiency-induced osteoporosis in female mice.	Inhibits oxidative stress, osteocyte senescence, and the senescence-associated secretory phenotype (SASP), promotes osteoblast-mediated bone formation, and suppresses osteoclast-driven bone resorption.	[182]			
**Immune System Aging and Chronic Inflammatory Conditions**	Protects intestinal function in aged mice and confers cytoprotective effects against d-galactose-induced cellular senescence.	Enhances intestinal NAD^+^ levels and activates SIRT3/SIRT6-mediated signaling pathways.	[72]	Enhances mitochondrial biogenesis and reduces low-grade inflammation	Upregulates PGC-1α, SIRT1, and TFAM; suppresses NLRP3 and caspase-1	[96]	Delays the progression of age-related hearing loss in CBA/CaJ mice.	Attenuates cochlear inflammation and suppresses apoptosis.	[183]
	Preserves intestinal function and extends lifespan in progeroid mice.	Increases NAD^+^; activates SIRT1 and NMNAT2/3; modulates Wnt/β-catenin signaling.	[184]				Inhibits osteoarthritis progression both in vitro and in vivo.	Activates SIRT6; inhibits NF-κB-dependent IL-1β signaling.	[146]
							Prevents radiation-induced gastrointestinal injury.	Suppresses ROS generation; reduces DNA and mitochondrial damage; inhibits apoptosis.	[185]
							Modulates palmitate-induced cell death.	Reduces MAPK cascade activity and exerts anti-inflammatory effects through modulation of IL-6 signaling.	[186]
**Cardiovascular and Vascular System Aging**	Confers comprehensive cardioprotection against doxorubicin-induced cardiotoxicity in male rats.	Reduces circulating BNP and LDH levels and restores mitochondrial biogenesis via the PGC-1α/NRF1/TFAM pathway.	[187]	Prevents oxidative damage in adult rat cardiomyocytes.	Reduces oxidative stress, mitochondrial dysfunction, and cell death in isolated cardiomyocytes.	[188]	Alleviates age-related cardiovascular dysfunction in mice and extends healthy life expectancy.	Increases Nrf2 protein levels and HO-1 translation in cardiac tissues, conferring antioxidant protection, inhibiting age-related ROS accumulation, and reducing inflammatory biomarkers in the heart and aorta.	[147]
	Attenuates doxorubicin-induced cardiotoxicity in male rats.	Attenuates cardiomyocyte apoptosis and cardiac fibrosis while suppressing oxidative stress, inflammation, and apoptotic signaling.	[189]	Prevents chronic heart failure in rat models.	Upregulates PGC-1α and TFAM to regulate mitochondrial biogenesis; increases NCLX expression to reduce ROS production, protect ΔΨm, and prevent mitochondrial Ca^2+^ overload.	[109]	Reduces mortality and lowers the risk of cardiovascular disease.		[130]
	Alleviates myocardial dysfunction in tail-suspension-induced cardiac impairment mouse models.	Replenishes NAD^+^ levels, upregulates Nrf2 protein expression, increases SOD activity, and decreases intracellular ROS and MDA accumulation.	[190]				Attenuates atherosclerosis in Ldlr^−/−^ mice fed a high-fat diet.	Downregulates NF-κB and Nrf2.	[191]
	Improves cardiac function and bioenergetic capacity in a mouse model of Friedreich's ataxia-associated cardiomyopathy.	Replenishes NAD^+^ and improves cardiac and extracardiac metabolic function as well as energy metabolism via SIRT3-dependent mechanisms.	[73]				Improves post-myocardial infarction cardiac remodeling and function in rats.	via S-glutathionylation through the NF-ĸB dependent Wnt5a-sFlt-1 pathway.	[192]
	Prevents doxorubicin-induced cardiotoxicity and preserves physical activity in mice.	Prevents apoptosis, oxidative stress, and inflammation, and suppresses p53- and promyelocytic leukemia nuclear body (PML-NB)-associated transcriptomic alterations.	[193]						
	Exerts acute cardioprotective effects in mouse models.	Enhances glycolysis-mediated cardioprotection, with downstream mechanisms including increased ATP production and improved tolerance to ischemia and/or reperfusion-induced acidosis.	[194]						
**Reproductive System Aging and Fertility Decline**	Reverses ovarian aging in rats.	Restores LH/FSH balance and mitochondrial dynamics; increases SIRT1 activity.	[71]	Improves premature ovarian failure in mice.	Enhances SIRT1- and PGC-1α-mediated mitochondrial biogenesis; inhibits ATM and p53 activation to reduce DNA damage-induced apoptosis.	[195]	Improves post-thaw semen quality in roosters.	Enhances total sperm motility, progressive motility, mitochondrial activity, and membrane integrity.	[196]
	Improves age-related decline of ovarian reserve in mice.	Reduces p16 levels in granulosa cells; upregulates CLPP and CTSD expression.	[197]	Prevents alkylating agent-induced ovarian dysfunction in mice.	Prevents mitochondrial oxidative damage and impaired biogenesis; promotes granulosa cell proliferation, inhibits granulosa cell apoptosis, and delays ovarian stromal cell senescence.	[198]			
				Alleviates ovarian ischemia-reperfusion injury in rats.	Increases SOD activity.	[199]

**Table 7:** Clinical trials on different compounds.

Compound	Subject	Administration method	Dosage	Dosage schedule	Side effect	Reference
NMN/NR	Healthy men and women aged 20-65 years	oral NMN	1250 mg/day	4 weeks	N/A	[200]
	Healthy men and women aged 40-65 years	oral NMN	300, 600, and 900 mg/day	60 days	N/A	[74]
	Healthy men aged ≥65 years	oral NMN	250 mg/day	6 or 12 weeks	N/A	[76]
	Healthy men and women aged 40-60 years	oral NR	100, 300, and 1000 mg/day	8 weeks	N/A	[201]
	Obese, insulin-resistant men aged 40-70 years	oral NR	2000 mg/day	12 weeks	N/A	[202]
PQQ	Healthy men and women aged 40-80 years	oral	21.5 mg/day	12 weeks	N/A	[111]
	Mild cognitive impairment, men and women aged ≥65 years	oral	20 mg/day	6 weeks	N/A	[112]
	Healthy men and women aged 50-70 years	oral	20 mg/day	12 weeks	N/A	[203]
EGT	Mild cognitive impairment, men and women aged 60-90 years	oral	25 mg/time; 3 times a week	1 year	N/A	[148]

### 6.1 Nervous system aging and neurodegenerative disorders

NMN/NR protects neuronal and neurovascular function through multiple pathways. In aged brain microvascular endothelial cells, NMN/NR restores NAD^+^ levels and promotes angiogenesis, helping prevent vascular cognitive impairment [69]. NMN/NR also mitigates cognitive dysfunction by reducing microglial activation and phagocytic behavior, thereby preventing myelin degradation following cerebral hypoperfusion [170]. Furthermore, NMN/NR coordinates antioxidant, anti-inflammatory, and anti-apoptotic responses through the Sirt1/AMPK/PGC-1α pathway, enhancing IL-10, SOD, and CAT levels while suppressing TNF-α, IL-6, and AGEs. This reduces cortical/hippocampal TUNEL + apoptotic cells, thereby safeguarding cognitive function and intestinal barrier integrity [172]. Additionally, NMN/NR maintains a youthful neurovascular phenotype by restoring NAD^+^ levels in neurovascular units and activating sirtuin-related transcriptional changes [62].

PQQ exerts neuroprotective effects by enhancing mitochondrial biogenesis and modulating neurotoxic signaling pathways. In a rotenone-induced Parkinson's disease model, PQQ promotes mitochondrial biogenesis in neurons and SH-SY5Y cells by activating AMPK [90]. In a d-galactose-induced aging model, PQQ was shown to reduce glutamate neurotoxicity and decrease hippocampal p-Tau levels via the GSK-3β/Akt signaling pathway, thereby improving cognitive function [171]. Additional studies demonstrate PQQ's protective effects against noise-induced and age-related hearing loss [110]. Clinical studies indicate that dietary PQQ supplementation improves memory, attention, judgment, and cognitive function in middle-aged and elderly individuals, while also enhancing mitochondrial biomarkers and brain metabolism in elderly subjects with mild cognitive impairment [111,112]. Collectively, these studies further underscore PQQ's unique value as a “mitochondrial quality control node” within the nervous system.

The role of EGT in the nervous system is primarily manifested as background control against chronic, low-level oxidative stress and inflammatory burden. EGT-rich edible fungi eliminate senescent neurons, representing a potential anti-aging intervention [144]. In d-galactose-induced senescence models, EGT protects mitochondrial function and enhances learning and memory by boosting T-SOD activity, reducing MDA levels, and modulating Nrf2/HO-1 and AMPK/SIRT1/PGC-1α pathways [137]. Clinical and preliminary studies indicate that long-term EGT intake is safe and delays cognitive decline in elderly individuals with mild cognitive impairment [148]. In the 5XFAD Alzheimer's disease model, EGT reduces oxidative stress and amyloid plaque accumulation while restoring glucose metabolism, demonstrating its potential to prevent neurodegenerative diseases [173].

### 6.2 Integumentary system aging (skin aging)

The protective effect of NMN/NR against skin aging is manifested through its remodeling of the fundamental biological state of skin fibroblasts. Transcriptomic studies reveal that NMN/NR significantly alters gene expression in skin fibroblasts, enhancing cellular responses associated with protein homeostasis, RNA metabolic regulation, and anti-apoptotic signaling. These changes are closely linked to NMN/NR 's ability to elevate intracellular NAD^+^ levels and activate sirtuin-related pathways, accompanied by enhanced autophagy. This suggests that NMN/NR may delay skin cell aging by improving intracellular homeostasis networks [68]. At the delivery modality level, studies utilizing small extracellular vesicles (sEVs) derived from mesenchymal stromal cells loaded with β-NMN further solidify this mechanistic understanding. These investigations demonstrate that NMN-sEVs effectively activate the NAD^+^/SIRT3 signaling pathway, inducing mitochondrial autophagy to enhance mitochondrial quality control and delay skin aging [174]. In UV-induced photodamage models, NMN/NR 's skin-protective effects are particularly pronounced. Research revealed that β-NMN significantly enhances skin barrier function in mice and effectively alleviates multiple UVB-induced photoaging features. NMN/NR inhibits phosphorylation in the MAPK signaling pathway, downregulates the expression of pro-inflammatory factors TNF-α and IL-6, as well as MMP-1 (a protein associated with wrinkle formation), while restoring the expression levels of hyaluronan synthases HAS-1 and HAS-2. This maintains skin structural integrity and hydration capacity [175].

Interestingly, PQQ's role in skin aging primarily manifests through its inhibition of oxidative stress-induced cellular senescence and apoptosis. In a human dermal fibroblast senescence model induced by UVA irradiation, PQQ demonstrated significant protective effects, with its mechanism closely associated with the anti-apoptotic SIRT1/Nrf2/HO-1 signaling pathway and the activation of SIRT6 [98,99]. This study indicates that PQQ enhances cellular tolerance to oxidative damage, thereby mitigating photo-induced senescence phenotypes. In genetically accelerated aging models, PQQ also exhibits significant skin-protective effects. In Bmi-1 knockout mouse models, PQQ treatment markedly improved skin aging phenotypes through mechanisms involving promotion of cell proliferation, suppression of aging-related factors p16, p19, and p53 expression, and significant enhancement of autophagy levels [58,[79], [80], [81]]. These results indicate that PQQ delays skin-aging processes associated with stem cell dysfunction by activating autophagy and inhibiting cellular senescence.

The anti-aging effects of EGT in the skin system primarily rely on its potent antioxidant and anti-inflammatory properties. In a UVB-damage model, EGT significantly mitigates fibroblast senescence by preventing the downregulation of the Nrf2/HO-1 pathway and HSP70 in keratinocytes [143]. EGT protects keratinocyte function by inhibiting ROS production and the cleavage of pro-apoptotic proteins such as caspase-8 and PARP. It also reduces the release of paracrine inflammatory factors, including IL-1β, IL-6, and TNF-α, thereby maintaining fibroblast collagen homeostasis and suppressing their senescent phenotype. In a UVA-induced human dermal fibroblast model, EGT similarly significantly reduced ROS production and exerted anti-aging effects by inhibiting the AP-1 signaling pathway and activating Nrf2-mediated antioxidant gene expression (including HO-1, NQO-1, and GCLC), with effects exhibiting clear dose- and time-dependent relationships [136]. It is notable that the anti-aging effects of EGT extend beyond the skin itself to its appendages. In a hair follicle aging model, EGT mitigated oxidative stress-induced damage to dermal papilla cells by activating the SIRT1/Nrf2 signaling pathway [57,58]. It reduced the secretion of inflammatory mediators such as IL-6, IL-1β, and TNF-α while enhancing the production of pigment-promoting and follicle function-maintaining factors, such as SCF and SDF1, thereby antagonizing natural hair follicle aging [57,58]. In addition, EGT demonstrated significant protective effects in both in vivo [143] and in vitro [150,151] UV-induced oxidative stress models. It reduced MMP levels, minimized collagen loss, and enhanced skin antioxidant capacity by activating the PI3K/Akt/Nrf2 signaling pathway. In mouse models, EGT decreased skin MDA levels and increased SOD activity; in cell models, it suppressed the proportion of β-galactosidase-positive cells associated with aging and reduced p16 and γ-H2A expression, further validating its inhibitory effect on skin cell aging [150,151].

### 6.3 Metabolic and endocrine system disorders

The effects of NMN/NR are primarily manifested in the overall “recalibration” of NAD^+^-dependent metabolic networks. In diet- and age-induced type 2 diabetes models, NMN/NR improves glucose tolerance and enhances hepatic insulin sensitivity by restoring tissue NAD^+^ levels. This mechanism partially relies on SIRT1 activation, thereby reshaping gene expression profiles associated with oxidative stress, inflammatory responses, and circadian rhythms [53]. This effect is not merely hypoglycemic; it enhances the metabolic system's adaptability to fluctuations in energy input by restoring the NAD^+^-sirtuin axis. Notably, NMN/NR 's metabolic regulatory effects exhibit context-dependent behavior in tumor settings: high-dose NMN/NR induces ferroptosis in lung adenocarcinoma cells via the NAM-mediated SIRT1-AMPK-ACC pathway, thereby inhibiting tumor growth [70]. This suggests NMN/NR may both restore metabolic resilience in normal tissues and amplify metabolic stress in tumor cells.

Unlike NMN/NR, which focuses on NAD^+^ supply, PQQ's core value in metabolic and endocrine systems lies in enhancing mitochondrial functional reserve and tolerance to lipid metabolism. PQQ-producing *Escherichia coli* strain Nissle 1917 has been shown to reduce oxidative stress and hyperlipidemia in aged rats while improving mitochondrial function, suggesting PQQ may influence metabolic status via the gut-metabolic axis [176]. In a high-fat diet-induced fatty liver model, PQQ improved lipid metabolism and enhanced hepatocyte tolerance to fat accumulation and oxidative damage by promoting mitochondrial biogenesis and strengthening antioxidant and anti-apoptotic capacities [177]. Furthermore, PQQ is recognized as a broad regulator of mitochondrial, lipid, and energy metabolism. Its systemic metabolic regulatory effects extend beyond liver and lipid dysregulation; it also mitigates radiation-induced mitochondrial damage in lung tissue via MOTS-c-dependent mechanisms [108] thereby further underscoring its capacity to maintain mitochondrial homeostasis under metabolic stress conditions.

The role of EGT in metabolic and endocrine systems tends toward long-term suppression of chronic oxidative stress and inflammatory conditions. Metabolic diseases are often accompanied by persistent ROS generation and activated inflammatory signaling, and this low-level, persistent stress state serves as a key driver of insulin resistance, endothelial dysfunction, and the progression of complications. In a high-glucose-induced endothelial aging model, EGT delays endothelial aging and improves endocrine-related vascular function by reducing ROS production, upregulating SIRT1 and SIRT6, and inhibiting p66Shc and NF-κB signaling [138]. In aging and metabolic studies, EGT has also been found to regulate cholinergic neurotransmission, tyrosine metabolism, fatty acid oxidation, and autophagy processes, contributing to systemic metabolic homeostasis and lifespan extension [149]. In a rat model of type 2 diabetes, l-ergothioneine alone or in combination with metformin improved diabetes-related kidney injury by activating the Nrf2 antioxidant pathway, upregulating HO-1 and NQO1, and suppressing NF-κB, TGF-β1, and fibronectin expression [131], highlighting its adjunctive value in preventing and controlling metabolic complications.

### 6.4 Musculoskeletal system aging

The primary function of NMN/NR is to restore NAD^+^-dependent muscle and bone metabolic capacity. Human research has provided direct evidence: in healthy elderly males, chronic oral NMN/NR supplementation significantly elevated blood NAD^+^ and its metabolite levels, accompanied by improved muscle function. This suggests that correcting NAD^+^ deficiency alone can alleviate age-related muscle dysfunction [76]. In bone tissue, NMN/NR similarly exerts protective effects by delaying cellular senescence. By elevating intracellular NAD^+^ levels, NMN/NR significantly mitigates TNF-α-induced senescence in human osteoblasts and reverses their diminished bone synthetic capacity. In vivo studies further demonstrate that NMN/NR not only prevents osteoporosis in ovariectomized mice but also significantly promotes fracture healing in osteoporotic mice [180]. These findings collectively point to a key characteristic: NMN/NR does not merely improve bone mass or muscle strength, but rather supports both highly energy-dependent processes, bone remodeling and bone repair, by restoring NAD^+^-dependent cellular metabolic activity.

PQQ's advantages in the musculoskeletal system are more focused on counteracting oxidative stress-driven degenerative structural damage. In intervertebral disc degeneration models, PQQ significantly suppressed oxidative stress, cellular senescence, and senescence-associated secretory phenotypes in the nucleus pulposus and annulus fibrosus of naturally aged mice [178]. It also blocked IL-1β-induced matrix degradation, ROS accumulation, and cellular senescence in human nucleus pulposus cells in vitro. Mechanistically, the Keap1-Nrf2-Wnt5a signaling axis was identified as the key pathway mediating its protective effects [178]. This finding highlights the high dependence of intervertebral disc degeneration on the integrity of the antioxidant defense system. At the skeletal muscle level, dietary supplementation with PQQ can delay the natural aging process and d-galactose-induced muscle aging in mice [103], effectively alleviating muscle atrophy and weakness by improving mitochondrial function and reducing oxidative stress and inflammation levels. In bone tissue, PQQ further demonstrates its capacity to regulate bone remodeling imbalance: it inhibits osteoclast activation and reduces bone resorption by activating the MCM3-Keap1-Nrf2 signaling axis, upregulating Fbn1 transcription, and decreasing RANKL production in the osteoblast lineage [63,64]. In estrogen-deficient models, PQQ significantly reduces bone loss and enhances bone strength by inhibiting oxidative stress, osteocyte senescence, and SASP, with effects comparable to exogenous estrogen [182].

The role of EGT in the musculoskeletal system is primarily focused on mitochondrial quality control and the fine-tuning of function, demonstrating unique advantages, particularly at the neuromuscular junction. In spinal muscular atrophy mouse models, EGT supplementation significantly improved animal phenotypes and survival rates. Its core mechanism lies in stimulating mitochondrial autophagy to clear dysfunctional mitochondria in the diaphragm, thereby alleviating severe mitochondrial dysfunction [179]. Under exercise and load stress conditions, EGT also exerts regulatory effects on muscle homeostasis: in high-intensity exercise models, it stabilizes erythrocyte membrane structure, reduces red blood cell fragility, and modulates blood cell counts, suggesting its role in mitigating exercise-induced oxidative and mechanical damage [181]. At the molecular level, EGT accumulates in muscle mitochondria post-exercise training and has been demonstrated to directly bind and activate 3-mercaptopyruvate sulfurtransferase, thereby enhancing mitochondrial respiratory function and improving exercise performance [145]. This characteristic makes EGT particularly suitable as a long-term supportive factor for maintaining muscle functional stability and stress adaptation capacity.

### 6.5 Immune system aging and chronic inflammatory conditions

Multiple research indicate that NMN/NR supplementation significantly elevates NAD^+^ levels in the intestinal tissues of aged mice and activates SIRT3-and SIRT6-mediated signaling pathways. This concurrently alleviates oxidative stress, suppresses inflammatory responses, and maintains epithelial barrier integrity. In aged mice and d-galactose-induced cellular senescence models, NMN/NR restored abnormally elevated inflammatory factors such as IL-6ST, IL-1A, and NF-κB1 to near-normal levels while correcting the disrupted expression of the tight junction protein claudin-1 [72], suggesting its action is not merely anti-inflammatory at a single point but rather rebuilds local immune homeostasis by restoring intestinal structure and immune coupling status. Further studies revealed that long-term β-NMN/NR supplementation not only extended the lifespan of prematurely aged mice but also significantly improved colonic function in aged mice. At the molecular level, NMN/NR upregulates SIRT1, NMNAT2, and NMNAT3 protein expression while suppressing abnormal P53 activation. More importantly, it maintains intestinal stem cell renewal capacity by activating Wnt/β-catenin signaling and upregulating Lgr5, thereby supporting long-term epithelial repair and immune homeostasis at the source [184]. Overall, these findings collectively demonstrate that NMN primarily delays the onset of immune aging by restoring NAD^+^-dependent epithelial renewal and immune regulatory capabilities.

The role of PQQ in immune aging is more focused on chronic low-grade inflammation driven by metabolic imbalance. In obesity-related chronic inflammation models, PQQ supplementation alone or in combination with atorvastatin simultaneously improves glucose tolerance, lipid profiles, and insulin indices. Mechanistic studies reveal that PQQ enhances energy status in metabolic cells by upregulating the transcriptional levels of PGC-1α, SIRT1, and TFAM, thereby boosting mitochondrial biogenesis and mtDNA content. Concurrently, it inhibits the activation of the NLRP3 inflammasome and Caspase-1, directly severing the amplification loop of the inflammatory cascade [96]. This characteristic suggests that PQQ not only corrects mitochondrial dysfunction, a key metabolic basis of chronic inflammation, but also suppresses inflammatory signaling, thereby reducing persistent immune system activation in aging and obesity.

The role of EGT in immune aging demonstrates its capacity to fine-tune the local chronic inflammatory microenvironment. In an age-related hearing loss model, EGT supplementation significantly slowed auditory function deterioration in CBA/CaJ mice, with its efficacy closely correlated to concurrent reductions in inflammatory responses and apoptotic levels within cochlear tissue [183], suggesting EGT delays immune-related sensory system aging by suppressing local inflammation. In arthritis, a prototypical chronic inflammatory disease, EGT significantly inhibits disease progression both in vivo and in vitro [146], further supporting its broad applicability in inflammation-driven tissue degeneration. In the context of intestinal inflammation, EGT's immunoprotective effects are particularly systemic: the ergothioneine hyaluronic acid gel (EGT@HA gel) effectively prevents radiation-induced enterocolitis by scavenging free radicals, inhibiting ROS production, and mitigating DNA and mitochondrial damage, thereby reducing apoptosis. At the tissue level, with prolonged gastrointestinal retention time, EGT@HA gel markedly reduced neutrophil infiltration and gut microbiota dysbiosis, alleviating radiation-induced tissue damage [185]. Furthermore, in a lipotoxicity-related inflammation model, EGT demonstrated marked protective effects against palmitate-induced C2C12 cell death, potentially through reduced MAPK cascade signaling activity and IL-6-related anti-inflammatory regulation [186].

### 6.6 Cardiovascular and vascular system aging

NMN/NR demonstrates systemic protective effects centered on NAD^+^ restoration across multiple cardiovascular aging and injury models. By elevating intracellular NAD^+^ levels, NMN/NR activates SIRT3-related pathways, thereby improving energy metabolism in cardiac and extracardiac tissues [73]. Interestingly, in an Adriamycin-induced cardiac toxicity model, NMN/NR 's effects extend beyond metabolic enhancement. It further inhibits NLRP3 inflammasome activation, increases GSH and SOD activity, reduces ROS and MDA levels, and downregulates caspase-1 and IL-1β activity, ultimately mitigating cardiomyocyte apoptosis and fibrosis [189,193]. Meanwhile, NMN/NR restores mitochondrial function and biogenesis by activating the PGC-1α/NRF1/TFAM axis, reduces BNP and LDH levels, and maintains myocardial integrity structurally and functionally [187]. In aging models and mechanically unloaded models like tail suspension, NMN/NR further sustains cardiac function by enhancing autophagy flux, upregulating Nrf2, and suppressing oxidative stress [190]. Under acute ischemic or acidic stress conditions, NMN/NR also rapidly boosts ATP production by promoting glycolysis, demonstrating its direct cardiac protective effects on a shorter timescale [194].

Compared with PQQ's role in the cardiovascular system, mitochondrial quality control is its primary focus rather than metabolic substrate supplementation. In isolated adult rat cardiomyocytes, PQQ directly reduces cell death by decreasing oxidative stress and maintaining mitochondrial membrane potential [188]. In vivo models of stress-induced cardiac remodeling, PQQ preserves PGC-1α and TFAM expression levels, thereby inhibiting myocardial hypertrophy and delaying progression to chronic heart failure [109]. Under complex stress conditions such as hypoxia, PQQ-containing combination formulations further stabilize mitochondrial structure and function, suggesting their potential to maintain cardiomyocyte homeostasis in multi-injury contexts [204].

EGT primarily participates in regulating cardiac and vascular aging by limiting oxidative stress and inflammatory propagation. Long-term consumption of EGT-rich edible fungi can upregulate Nrf2 and HO-1 protein levels in cardiac tissue, reduce age-related accumulation of ROS, and suppress inflammatory marker expression [147]. Population studies demonstrate that elevated plasma EGT levels are significantly associated with reduced cardiovascular disease incidence and all-cause mortality, providing clinically relevant evidence of its cardiovascular protective effects [130]. In acute myocardial infarction models, EGT downregulates MCP-1, p65, p-p65, sFlt-1, and GLRX expression, thereby improving post-infarction myocardial remodeling and functional recovery [192]. Furthermore, EGT inhibits atherosclerotic progression in high-fat diet-fed Ldlr^−/−^ mice, further demonstrating its capacity to suppress vascular pathological alterations [191].

### 6.7 Reproductive system aging and fertility decline

The role of NMN/NR in ovarian aging intervention has been demonstrated to remain SIRT-related and capable of remodeling the follicular microenvironment. In a middle-aged rat model, NMN/NR significantly alleviated mitochondrial stress by inhibiting excessive mitochondrial fission in the ovaries, restoring the fission-fusion balance, and correcting aging-related folliculogenesis abnormalities[71]. This process is accompanied by enhanced SIRT1 activity and restored LH/FSH ratios, suggesting NMN/NR not only improves local energy metabolism but also exerts systemic regulatory effects on the reproductive endocrine axis. In long-term intervention models, NMN/NR further demonstrated significant improvement in age-related decline in ovarian reserve by enhancing mitochondrial autophagy in granulosa cells [197]. NMN/NR markedly reduced expression of the senescence marker p16 in granulosa cells while upregulating CLPP and CTSD levels, suggesting it maintains follicular functional integrity by promoting damaged mitochondrial clearance through regulation of mitochondrial-lysosomal protein homeostasis.

The protective role of PQQ in ovarian aging primarily manifests as promoting mitochondrial renewal and inhibiting apoptosis induced by DNA damage. In models of premature ovarian failure, co-administration of PQQ with human mesenchymal stem cell-derived mitochondria significantly enhances SIRT1/PGC-1α-mediated mitochondrial biogenesis while suppressing abnormal activation of the ATM and p53 signaling pathways. This reduces DNA damage-induced granulosa cell apoptosis and improves ovarian function [195]. Moreover, dietary PQQ supplementation significantly prevented alkylating agent-induced ovarian dysfunction, with protective effects manifested as enhanced granulosa cell proliferation, suppressed apoptosis, and reduced ovarian stromal cell senescence [198]. In acute injury models, intraperitoneal injection of PQQ markedly alleviated histological damage and biochemical abnormalities caused by ovarian ischemia-reperfusion, further supporting its direct protective role in ovarian mitochondrial and cellular survival under stress conditions [199].

EGT has been relatively understudied in reproductive system research, but existing evidence indicates it protects sperm function through antioxidant mechanisms. In a rooster semen cryopreservation-thawing model, supplementing culture medium with 5-10 μM ergotamine significantly improved total motility and progressive motility of thawed sperm while enhancing mitochondrial activity and membrane integrity [196]. This result indicates that ergothioneine may improve sperm quality by mitigating mitochondrial dysfunction through scavenging ROS generated during cryopreservation-thawing.

## 7 Limitations and translational challenges

Although NMN/NR, PQQ, and EGT have consistently demonstrated potential to improve mitochondrial dysfunction associated with aging across multiple in vitro and animal models, their translation to clinical and population levels remains limited. Notably, these limitations stem not primarily from insufficient efficacy of individual molecules, but rather from challenges in defining safe exposure boundaries for combined applications, controlling dosage ratios and delivery methods, and addressing uncertainties regarding potential interactions.

In basic research, NMN/NR, PQQ, and EGT typically produce clear biological effects when administered intraperitoneally or at relatively high dietary supplement doses. However, the feasibility and long-term safety of these administration strategies in humans require careful evaluation. Regulatory bodies have begun addressing these concerns. According to the European Food Safety Authority (EFSA) document PC-1537 published on July 18, 2025, NMN is proposed for inclusion in the Novel Foods Register, with a recommended maximum intake of 500 mg/day for the general adult population. EFSA also proposed daily upper intake limits for PQQ and EGT at approximately 20 mg/day [205] and 30 mg/day (EFSA Panel on Dietetic Products et al., 2016), respectively. Notably, existing human studies have to some extent expanded the safety range, which could be seen in Table 6. Multiple clinical trials indicate that even when NMN oral doses are increased to 900 mg/day for 60 consecutive days, it demonstrates good safety and tolerability in healthy individuals aged 40-65 years [74]; higher doses (1250 mg/day for 4 consecutive weeks) also showed no significant adverse reactions in healthy subjects aged 20-65 [200] Similarly, healthy Japanese men and women aged 40-80 years showed significant improvements in memory, attention, and judgment after 12 weeks of daily supplementation with 21.5 mg of PQQ, with more pronounced effects observed in middle-aged and elderly individuals [111]. Regarding EGT, a 1-year study demonstrated that daily supplementation with 25 mg was not toxic in elderly individuals with mild cognitive impairment, accompanied by improved cognitive function and slowed neuronal damage [148]. However, it must be emphasized that currently available human studies suffer from limited sample sizes, relatively short intervention periods, and a focus predominantly on healthy or sub-healthy populations. This implies a lack of systematic toxicological evaluation for long-term combined supplementation, chronic disease contexts, or individuals with abnormal metabolic burdens. Therefore, while existing evidence generally supports the safety of all three within recommended or slightly elevated dosage ranges, long-term exposure risks and potential population variations remain significant barriers to translational application.

Beyond single-ingredient dosages, the ratio of NMN/NR, PQQ, and EGT also represents a critical uncertainty limiting their clinical translation. Currently, formulation designs across different application scenarios remain fragmented. One patented formula for skin anti-aging proposes an equal 1:1:1 combination of the three ingredients[161], while anti-aging and anti-inflammatory patents allow ratios ranging from 20 to 1000:1-100:1-100[160]. Such broad ratios have also appeared in some commercial dietary supplements. For example, the Japanese product “Hama Shoku NMN36000” contains 200 mg NMN, 20 mg PQQ, and 2.5 mg EGT per capsule, with a recommended daily intake of 2-3 capsules; Another combined product, “Health Life® NMN Ultimate 27,000 mg + PQQ 90s,” contains 300 mg of NMN and 20 mg of PQQ per capsule, with a recommended daily intake of 1-2 capsules with meals. The formulation ratios for such products may be based more on empirical observations or market strategies than on systematic pharmacodynamic or metabolic synergy evidence.

The route of administration and delivery system also profoundly influences the translational potential of these three approaches. For instance, in mouse experiments, oral NMN/NR administration showed no significant adverse effects, whereas intraperitoneal injection led to elevated oxidative stress in sperm tissue [78]. This phenomenon indicates that certain commonly used laboratory administration methods may not be directly extrapolated to long-term application scenarios. In recent years, multiple novel delivery strategies have been explored to enhance bioavailability and reduce potential side effects. For example, an NMN/NR nanoparticle delivery system based on ovalbumin and fucoidan can increase NAD^+^ levels in vivo by approximately 1.34-fold [206]; while mitochondria-modulating nanoliposome systems (ECG-Lipo) have been demonstrated to co-deliver EGT and lipophilic coenzyme Q10, significantly improving skin permeability and cellular repair capacity [58,[79], [80], [81]]. From a translational medicine perspective, this discrepancy highlights a core issue: the synergistic effects of NMN/NR, PQQ, and EGT are not only constrained by dosage levels but are also coupled and regulated by dosage ratios and administration routes.

In combined application situations, the potential interactions among NMN/NR, PQQ, and EGT pose a difficult-to-predict risk dimension. In the field of nutritional and functional supplement research, when two or more bioactive compounds are co-administered, their overall biological effects do not necessarily exhibit linear summation but may manifest in three distinct patterns: additive, synergistic, or antagonistic[207]. For NMN/NR, PQQ, and EGT, existing experimental and clinical evidence primarily relies on single-molecule or dual-component models. Although patented and commercial products have attempted to combine these three compounds[160,161]. These practices have not fully demonstrated systematic pharmacodynamic and mechanism validation. While this uncertainty is not abstract from a biological mechanism perspective but has clear theoretical foundations, the safety and efficacy of their combined supplementation still require systematic experimental and clinical translational validation.

## 8 Conclusion

The triaxial synergistic model of NMN/NR, PQQ, and EGT suggested in this review integrates NAD^+^-dependent metabolic support, mitochondrial remodeling, and redox buffering into a unified anti-aging framework. Existing preclinical and preliminary clinical evidence indicates that these three compounds possess potential protective effects across cardiovascular, neurological, metabolic, musculoskeletal, immune, skin, and reproductive systems. Nevertheless, systematic research is still needed to optimize dosages, develop combination strategies, and refine delivery systems. Future studies should focus on multi-target mechanism validation, long-term safety assessments, and biomarker-guided precision interventions to advance this tri-axial strategy toward clinical translation, ultimately achieving systemic anti-aging interventions targeting mitochondria.

## Funding

This research is financially supported by 10.13039/501100001509Royal Society of New Zealand 10.13039/100027448Catalyst Seeding Fund, 10.13039/100018696Health and Happiness HK Ltd., EZZ Life Science Pty Ltd., and Oxyenergy Ltd.

## CRediT authorship contribution statement

**Yidan Sun:** Conceptualization, Data curation, Investigation, Methodology, Visualization, Writing – original draft. **June-Chiew Han:** Methodology, Resources, Supervision, Writing – review & editing. **Kenneth Tran:** Conceptualization, Resources, Supervision, Writing – review & editing. **Toan Pham:** Conceptualization, Investigation, Project administration, Supervision, Writing – review & editing. **Qiang Zhou:** Conceptualization, Funding acquisition, Resources, Supervision, Writing – review & editing. **Jun Lu:** Conceptualization, Funding acquisition, Resources, Supervision, Writing – review & editing.

## Declaration of competing interest

The authors have declared no conflict of interest.

## Data Availability

No data was used for the research described in the article.
